# Copper-Modified Polymeric Membranes for Water Treatment: A Comprehensive Review

**DOI:** 10.3390/membranes11020093

**Published:** 2021-01-28

**Authors:** Andreina García, Bárbara Rodríguez, Hugo Giraldo, Yurieth Quintero, Rodrigo Quezada, Natalia Hassan, Humberto Estay

**Affiliations:** 1 Mining Engineering Department, FCFM, Universidad de Chile, Santiago 8370451, Chile; 2 Advanced Mining Technology Center (AMTC), Universidad de Chile, Santiago 8370451, Chile; hugo.giraldo@amtc.cl (H.G.); yurieth.quintero@amtc.uchile.cl (Y.Q.); rodrigo.quezada.m@uchile.cl (R.Q.); humberto.estay@amtc.cl (H.E.); 3 Programa Institucional de Fomento a la I+D+i, Universidad Tecnológica Metropolitana, Ignacio Valdivieso 2409, San Joaquín, Santiago 8940577, Chile; nhassan@utem.cl

**Keywords:** copper nanomaterials, polymeric membranes, biofouling, water treatment, nanocomposites

## Abstract

In the last decades, the incorporation of copper in polymeric membranes for water treatment has received greater attention, as an innovative potential solution against biofouling formation on membranes, as well as, by its ability to improve other relevant membrane properties. Copper has attractive characteristics: excellent antimicrobial activity, high natural abundance, low cost and the existence of multiple cost-effective synthesis routes for obtaining copper-based materials with tunable characteristics, which favor their incorporation into polymeric membranes. This study presents a comprehensive analysis of the progress made in the area regarding modified membranes for water treatment when incorporating copper. The notable use of copper materials (metallic and oxide nanoparticles, salts, composites, metal-polymer complexes, coordination polymers) for modifying microfiltration (MF), ultrafiltration (UF), nanofiltration (NF), forward osmosis (FO) and reverse osmosis (RO) membranes have been identified. Antibacterial and anti-fouling effect, hydrophilicity increase, improvements of the water flux, the rejection of compounds capacity and structural membrane parameters and the reduction of concentration polarization phenomena are some outstanding properties that improved. Moreover, the study acknowledges different membrane modification approaches to incorporate copper, such as, the incorporation during the membrane synthesis process (immobilization in polymer and phase inversion) or its surface modification using physical (coating, layer by layer assembly and electrospinning) and chemical (grafting, one-pot chelating, co-deposition and mussel-inspired PDA) surface modification techniques. Thus, the advantages and limitations of these modifications and their methods with insights towards a possible industrial applicability are presented. Furthermore, when copper was incorporated into membrane matrices, the study identified relevant detrimental consequences with potential to be solved, such as formation of defects, pore block, and nanoparticles agglomeration during their fabrication. Among others, the low modification stability, the uncontrolled copper ion releasing or leaching of incorporated copper material are also identified concerns. Thus, this article offers modification strategies that allow an effective copper incorporation on these polymeric membranes and solve these hinders. The article finishes with some claims about scaling up the implementation process, including long-term performance under real conditions, feasibility of production at large scale, and assessment of environmental impact.

## 1. Introduction

Globalization, growing population, insufficient water sources, industrialization, and global warming have contributed to the growing demand of water resources and their scarcity, causing a global concern [1,2].

**Figure 1:**
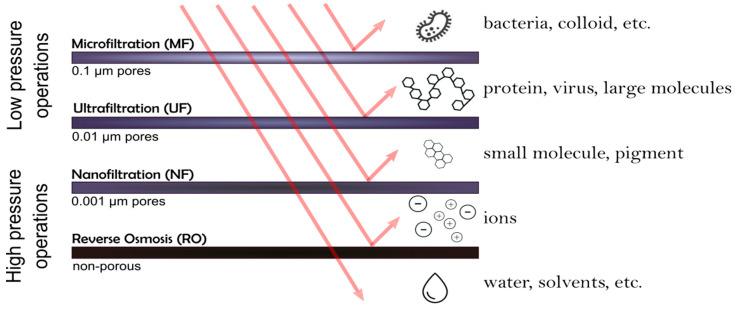
Schematic representation of membranes-based separation process.

Water consumption by 2030 is expected to increase above the current level; it will be impossible to obtain it from traditional sources of freshwater [3]. Securing and allocating sufficient water resources has, thus, become one of the current major global challenges [3,4]. In this regard, scientific and technological approaches to find alternative sources to obtain fresh water have been the center of attention. The development of suitable methods to obtain freshwater, from saltwater and water reuse, has driven many researchers in the last years. In this regard, membrane-based processes are key components, dominating the field of water purification. These membrane-based processes require a pressure difference that work as a driving force. They can be grouped into four categories, according to the molecules or particles size to be separated and, therefore, according to the membrane pore size. Based on these properties, they have the ability to remove different contaminants, as shown in Figure 1. The main membranes categories are: microfiltration (MF), ultrafiltration (UF), nanofiltration (NF) and reverse osmosis (RO) [5].

The membrane performance mainly depends on the operational conditions and the material properties. In this regard, the development of novel membrane materials plays a central role in advancing the field of membrane technology [2]. In the last twenty years, novel research has focused on the development of synthetic membranes with improved properties for different applications associated with the water treatment process. For this purpose, different proposals by using organic material (polymers) or inorganic material (ceramics) were synthesized. Polymeric membranes have been widely used in comparison with ceramic membranes due to their better chemical stability, mechanical strength and the lower cost in terms of water produced [6,7]. Finally, it is easier to modify polymeric materials rather than ceramic membranes, so their application and advantages are feasibly expanded. Breakthrough effects that have been reported in the production of polymer membranes in the field of water and wastewater treatment include fouling mitigation, improvement of permeate quality and flux enhancement [8,9]. In addition, challenges to enhance long-term membrane stability, reliability, and cost efficiency have also been highlighted [10,11].

Regarding membrane fouling, it is the most critical point within membrane technologies. Although it is an inevitable obstacle in the process, it causes deterioration of the membrane performance and, consequently, higher operation and maintenance costs for cleaning and replacing [12,13,14]. Membrane fouling can be broadly categorized into three types: inorganic fouling, organic fouling and biofouling. Inorganic fouling is caused by the scale formation at the membrane surface, whereas organic fouling by the deposition of organic foulants (e.g., surfactants) on the membrane surface [10,15]. Biofouling is caused by the attachment and proliferation of microorganism communities to the membrane surface, which eventually form a biopolymer matrix or complex structure, regarded as a biofilm on the membrane surface [14].

Thus, fouling mitigation on membrane technology is necessary and must be solved with innovative and sustainable approaches. Several strategies have been studied to overcome these problems, such as: (1) optimization of the operation process; (2) use of pre-treatment of feed water; and (3) improvement of membrane properties by chemical surface modification [14,16]. 

In this last point, novel membranes with inherent anti-fouling capabilities for different water treatment applications, such as microfiltration, nanofiltration, ultrafiltration, forward and reverse osmosis have been reported [12,17,18,19,20]. Composite membranes as a result of the addition of different nano-materials into the polymer matrix have been an alternative that has attained much attention in antifouling membrane fabrication [14,21,22]. In general, the major factors influencing membrane fouling are associated with the physicochemical properties of membrane surface [21,23,24], such as hydrophilicity, roughness and electrostatic charge [17,25,26]. Herein, research related to these chemical surface modification has involved the introduction of hydrophilic layer mainly to favor the water permeability; the reduction of surface roughness since that biofoulants are entrapped in membranes with rougher topologies; and the improvement of charge property to favor the electrostatic repulsive force between the charged membrane surface and the foulant [14,21,27,28]. 

In addition, the combination with antimicrobial materials into membranes also becomes an innovative potential solution to biofouling control mainly. For example, silver (Ag), titanium dioxide (TiO_2_), graphene oxide (GO), iron (Fe), copper (Cu), Zinc (Zn), metal organic frameworks (MOFs), whose antibacterial properties are well known, have been incorporated into a wide variety of water filtration membranes [10,22,29,30,31,32,33,34,35,36,37]. 

There is particular interest in the use of copper-based materials in recent years. Copper has attractive characteristics: it has an excellent antimicrobial activity, it is one of the most toxic metals to heterotrophic bacteria in aquatic environments [29,38], and it has recently been registered by the US Environmental Protection Agency (EPA) as the first antimicrobial element [39]. Its effectiveness to inhibit the growth of pathogenic microorganisms make it an ideal candidate to be an active biocide agent. Consequently, it has been extensively used in the design of antibacterial polymeric materials for biomedical devices, food processing equipment, antifouling paints, among others [9,10,14,21,22]. In fact, it is particularly attractive due to its high natural abundance and low cost. 

Another relevant feature is that multiple cost-effective synthesis routes are used to obtain copper based materials with tunable characteristics. For example, nanoparticles, composites, metal-polymer complex, and coordination polymers favor their incorporation into polymeric membranes [27,40,41,42,43]. 

**Figure 2:**
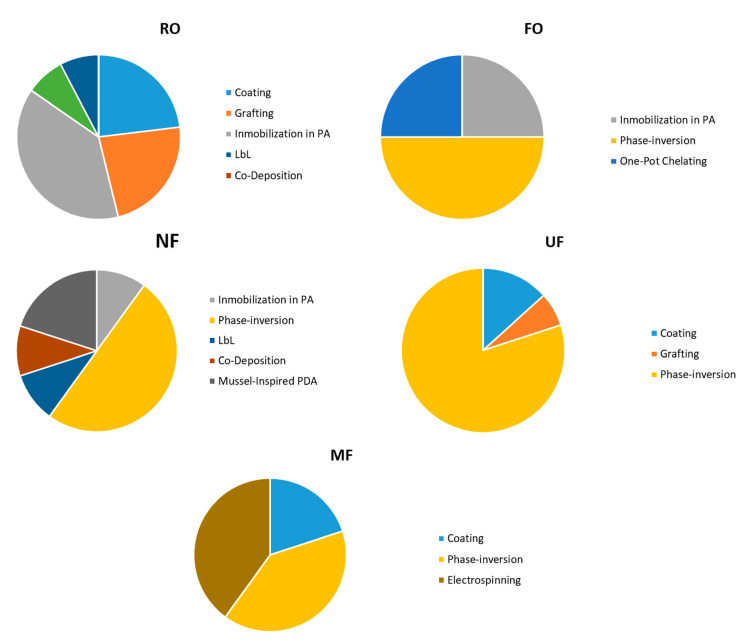
Distribution of the different techniques used for modified membranes with copper. The diagrams are plotted using the data presented in articles [14,27,28,32,36,40,42,43,44,45,46,47,48,49,50,51,52,53,54,55,56,57,58,59,60,61,62,63,64,65,66,67,68,69,70,71,72,73,74,75,76,77,78,79,81,82,83,84,85,86,87].

During the last 10 years, the notable use of copper-based materials for modifying MF [44,45,46,47,48], UF [28,49,50,51,52,53,54,55,56,57,58,59,60,61,62], NF [42,63,64,65,66,67,68,69,70,71], forward osmosis (FO) [72,73,74,75] and RO membranes [14,21,27,32,76,77,78,79,80] has attained much attention by the scientific community (according to the search strategy and eligibility criteria used, see Supplementary Materials, Prisma checklist, Prisma flow diagram and SRProtocol). The combination of these polymeric membranes with copper materials such as metallic and oxides nanoparticles, salts, composites, metal-polymer complex, and coordination polymers, offer a solution against biofouling formation, and, in addition, promote the improvement of other membrane properties. For these reasons, different membrane modification routes have been explored, including the incorporation of copper-based materials during the synthesis process of the membrane or the surface membrane modification using physical and chemical surface modification techniques (See Figure 2) [4,11,12,27,44,64,71,75,76,77]. Thus, the copper influence on membrane properties beyond the anti-biofouling effect, the impact of the modification, and the used method on the overall membrane properties and performance are aspects to be analyzed. Moreover, the modified membrane stability and copper leachability to operate for longer times under typical hydrodynamic conditions of a water treatment system must be also considered. These areas should be specifically addressed in order to make the industrial application of these modified membranes possible.

Based on the above, this is a comprehensive review about the progress of modified membranes for water treatment by incorporating copper. Advantages and limitations on the reported modifications for each membrane type (RO, FO, NF, UF and MF) and the used methodologies are critically presented. Thus, the drawbacks in the incorporation of copper into membrane matrices are identified. Equally, strategies that might allow an effective copper incorporation on these polymeric membranes through membrane modification procedure improvements are also offered. Furthermore, perspectives about a scale up implementation considering aspects such as long-term performance under real conditions, feasibility of production at large scale, and assessment of environmental impact by the use of copper are presented. Finally, the shortfalls and future perspectives of these modified polymer membrane technology are highlighted and offer insights for further progress in the field of water treatment and desalination using these copper-modified polymeric composite membranes.

## 2. Overview of Copper

### 2.1. Properties and Toxic Mechanism 

Copper is a transition metal element that can be found in different natural underground or rock deposits. This element shows excellent affinity with sulfur and is one of the most common components of sulfide ores such as pyrite, chalcopyrite, cuprite (oxides), and carbonates (malachite) [88,89]. This material has excellent properties: it is highly ductile and malleable, with high thermal and electrical conductivity. It can also be indefinitely recycled, and it can form alloys to improve mechanical properties, corrosion, and oxidation resistance, allowing extensive applications [90]. Moreover, copper has mainly been used due to its effectiveness as an antimicrobial material. Different research lines have thoroughly explored copper given its capacity to inactivate fungi [91], bacteria [92,93], viruses [94,95], parasites [96], and algae [91]. The ranking of pure metal cytotoxicity from most potent to least potent can be presented as follows: Cu > Al > Ag > V > Mn > Cr > Zr > Nb > Mo > Ti [97].

Overall, the efficacy of the microbicide effect of copper depends on several factors such as its physical form (bulk, nanoparticle, ions, etc.), its chemical state (elemental, copper oxide, etc.), concentration, wet or dry application form, temperature and humidity, and presence of buffer, among others. Copper toxicity can occur through two mechanisms: (1) direct contact killing, which depends on the proximity between the microorganism and Cu-containing surfaces, and (2) toxic effects induced by copper ions produced by copper dissolution [98,99,100].

It is known that the already mentioned parameters are clearly related to the contact killing effect of copper onto microorganisms. For example, high copper content, high temperature of application and the wet or dry condition to use it are the main factors that influence the killing of bacteria. Dry conditions have antimicrobial effectiveness in a few minutes, while wet conditions take several hours, which raises interesting questions about the contact killing mechanism [98]. Regarding the temperature of application, several works have demonstrated the effectiveness of the bacterial “contact killing” under high temperature and high humidity (37 °C-100% relative humidity) [29,93,98,101]. In addition, contact interactions strongly depend on the size of copper particles. It is well-known that nanoparticles show more serious toxicity than bulk species [99,102]. Small particles have greater interaction with biomolecules, which has a direct impact on their biocide capacity. Moreover, small copper particles increase the cellular uptake, where copper causes damage due to the interaction with intracellular molecules such as proteins and DNA [100].

Several studies revealed that the primary mechanism by which copper ions exert toxicity to bacteria is through depolarization of the cellular membrane [100,103]. This mechanism is led by the reduction of the electric potential because the Cu ions bind to the negatively charged domains in the bacteria cell membrane. The consequently depolarization causes cellular membrane rupture once the potential difference drops to zero [103]. Copper ions can affect the cell envelope of Gram-negative and Gram-positive bacteria, because they can bind to the peptidoglycans, carboxylic groups, or lipopolysaccharides of the outer membrane. The membrane depolarization of bacteria, thus the damage, depends on the bacterial morphology [104]. Alternatively, copper ions can also interact with biomolecules inside the cells, displacing metal-binding protein sites, and reducing or inactivating its activity [100].

Both mechanisms, contact killing and ion toxicity, lead to an increase in the oxidative stress of the cell membrane through the production of reactive oxygen species (ROS) [85,100,105]. Copper can impede the development of bacterial biofilm on a surface acting as a catalyst for redox reactions involving ROS. For example, redox cycling between Cu^2+^ and Cu^1+^ can generate the production of highly reactive hydroxyl radicals, which can subsequently damage all cell components of the biofilm, including lipids, proteins, DNA and other biomolecules [106]. DNA damage is a secondary effect of ROS generation by copper nanoparticles (NPs), as they can directly bind to domains in the DNA that impair their activity [107]. 

Likewise, copper nanoparticles and Cu ions can catalyze Fenton chemistry *in vitro*, generating the reactive hydroxyl radicals that participate in several reactions, oxidizing proteins, and lipids. The Fenton reaction is given by the following chemical reactions (1):

(1) $${\mathrm{Cu}}^{+}+H_{2}O_{2}\to {\mathrm{Cu}}^{+2}+\mathrm{OH}+{\mathrm{OH}}^{˙},$$

(2) $$2{\mathrm{Cu}}^{2+}+2\mathrm{RSH}\;\to 2{\mathrm{Cu}}^{+}+\mathrm{RSSR}+2H^{+},$$

(3) $$2{\mathrm{Cu}}^{+}+2H^{+}+O_{2}\to 2{\mathrm{Cu}}^{+2}+H_{2}O_{2},$$

Reactions (2) and (3) demonstrate how copper ions can lead to depletion of sulfhydryls, such as in cysteines or glutathione. This causes protein damage and increases the oxidative stress in the microorganism that finally leads to cell apoptosis [100]. 

Copper NPs have been reported to be around fifty times less toxic than the ionic form in mammalian cells. However, to bacteria, copper NPs are more lethal than ionic forms due to the above-mentioned capacity of contact killing and rapid dissolution in comparison to the bulk. Overall, copper nanoparticles are highly toxic against a wide variety of bacteria (gram positive and gram negative) and fungus. This is possible due to their favorable surface-to-volume-ratio, generally killing cells by diverse mechanisms previously mentioned such as membrane disruptions, blocking biochemical pathways, complex formations with proteins, and DNA damages [108]. However, a different bactericidal behavior has been observed in copper metallic (Cu-NPs) with respect to copper oxide (CuO-NPs) nanoparticles, revealing the impact of the type of copper chosen for this purpose. For instance, the oxide-state of copper in CuO-NPs limits its dissolution capacity compared to Cu-NPs, due to the less soluble surface of CuO. On the contrary, elemental Cu-NPs have a rapid initial dissolution that can decrease in prolonged exposure [85,105]. Accordingly, compared to copper salts, both NPs exhibit great ROS production, even at low concentrations of dissolved copper. However, in the case of Cu-NPs, interaction of the nanoparticles with the membrane of bacteria is key to ensure ROS-induced toxicity [85,109]. When Cu-NPs are incorporated in polymeric membranes, they could diminish ROS production due to the reduced direct contact with bacteria. Authors have also found that copper salts can be completely dissolved in short periods of time, yet they cannot reach greater levels of ROS production as Cu-NPs and CuO-NPs [109]. Thus, the significant influence of copper type on their ion release capacity and the ROS production should be considered because these are key parameters in the toxicity triggered by different copper types.

Another crucial aspect for the bacteria-killing process is related to the proximity of microbes to Cu-containing surfaces. In the presence of copper ions, complete cell death should occur. However, if an inert polymer covers the copper surface, there is a reduction of copper exposure and, as consequence, a copper ion release, causing the reduction of the bacterial death [110]. 

Moreover, in order to obtain a significant antimicrobial effect, a minimum inhibitory concentration (MIC) and minimum bactericidal concentration (MBC) of copper in microorganisms are required. These vary depending on the type of bacteria and form of copper. For example, for *E. coli*, the MIC of ionic copper is estimated around 2.25 µM [111]. In the case of nanoparticles, Table 1 shows the MBC of Cu and CuO-NPs per each type of bacteria.

Another consideration in the antimicrobial effect of copper is the presence of buffers or contaminants in the chemical environment. These play an essential role in copper antimicrobial activity. It was demonstrated that Tris-Cl buffer induced a 10-50-fold faster copper ion release compared to phosphate-buffered saline (PBS). Furthermore, copper ions and H_2_O_2_ present a synergistic action in *E. coli* killing in the presence of 4-(2-hydroxyethyl)-1-piperazine ethanesulfonate (HEPES), provoking catalysis of hydroxyls radicals release [113].

Finally, it is also important to consider that several mechanisms have been discussed related to the defense of bacteria in the presence of copper. These mechanisms are not entirely understood, because there is no evidence regarding a unique alternative to protect them from copper. As an example, in Gram-negative bacteria, such as *E. coli*, the *CopA,* an ATP-driven copper pump that expels copper (I) from the cytoplasm into the periplasmic space. There, it can become oxidized by the multi-copper oxidase CueO, which is involved in copper detoxification. This enzyme can also oxidize catechol siderophores, and the resulting pigments can capture copper. In the case of Gram-positive bacteria, only *CopA-type* copper transporters are present, since these kinds of bacteria do not have a periplasmic space and an outer membrane. Further research is required to understand how bacteria handle copper [103,114].

### 2.2. Copper-Based Materials and Relevant Features 

Almost 300 different forms of Cu-based materials were registered as antimicrobial products by EPA. For example, Cu/metals/alloys and metal substrates surface-modified with Cu, composites of Cu with polymers and glass, nonmetal substrates surface-modified with Cu, and superhydrophobic surfaces containing Cu have been widely used as cheap and effective materials for sterilizing, textiles and also human tissues for centuries. Moreover, their application in different fields such as in electronics, thermal energy, catalysis, photonics, biosensors and optoelectronics have been reported [25,115].

There is evidence of a particular interest in producing copper nanoparticles. The synthesis of different metallic and metal oxide copper nanoparticles have been widely studied. Copper NPs can be obtained by several strategies that include physical and chemical methods. For physical methods, the use of sophisticated equipment and technology is necessary, which makes them a relatively complex process. In the case of chemical methodologies, several strategies can be found in the literature due to their ease of control, simplicity of operation, limited equipment requirement and high quality of particles. Chemical methodologies, such as wet chemical reduction [116], reverse micelles [117], electrochemical and sonoelectrochemical techniques [118], vapor deposition [119], laser irradiation [120], thermal decomposition [121], thiol-induced reduction and microemulsions have been reported. In all these cases, it is very important to control the morphology, particle size and shape, surface charge and physicochemical properties of the synthesized nanoparticles [116]. 

Thus, the characteristics and properties of copper nanoparticles can be treated and controlled during their synthesis and adapted to be added on any solid surface, such as polymeric membranes. In general, the characteristics of the nanoparticles (size and shape, among others) can be dependent on the precursor [122]. For this reason, their choice is fundamental to obtain the desired features. Thus, copper nanoparticles often entail the reduction of Cu (I) or Cu (II) sources. Copper sulfate (CuSO_4_), copper acetylacetonate, copper chloride (CuCl_2_), or copper nitrate (Cu(NO_3_)_2_) have been used as a precursor. For wet chemical techniques, commonly used reducing agents are hydrazine, sodium borohydride, ascorbic acid, glucose, and 1,2-hexadecanediol, among others [123]. Several capping agents have been employed to stabilize the nanoparticles and control particle size [26]. Moreover, these agents could condition the surface chemistry of the nanoparticle to favor a specific functionality and impact on their properties such as hydrophilicity and shape [120,121]. In addition, for Cu-NPs, the most important challenge for these kinds of studies is to synthesize a stable Cu-NPs, which can be due to rapid oxidation to Cu^+2^ provoked by air or the aqueous media [124]. Therefore, the methodology to obtain these kinds of nanoparticles are performed in non-aqueous media and under inert atmosphere (argon, nitrogen) [26]. 

The size, shape and the surface chemistry of copper nanomaterials to be incorporated in the polymer membrane could exert tremendous impact on the membrane properties [125,126]. Thus, the incorporation of copper nanomaterials and their used synthesis agents can influence on surface properties of the modified membrane. Some benefits sought are related to the increase of the hydrophilicity, the reduction of surface roughness, and the improvement of charge property to favor the foulant reject from the modified membrane surface. For instance, CuO-NPs have showed hydrophilic character, which means that these oxide nanoparticles could improve in the surface hydrophilicity and/or the water flux of the modified membrane better than hydrophobic Cu-NPs [127]. Other aspects such as the size and their shape could also influence the surface-modified membrane, having an impact in the membrane performance. For instance, a different shape changes the exposed crystal facets and hence, the atomic arrangements in each facet could also have an intense effect on its surface properties. Moreover, an increase in surface membrane roughness can be influenced by the size of the incorporated nanoparticles [14,21,22,27]. Finally, the membrane surface charge could be altered after modification attributed to the coverage of the membrane surface by positive or negative charged copper NPs [32]. Thus, the control of size, shape and the surface chemistry of copper nanomaterials to be used in membrane modification are important aspects to be considered and are mainly dependent on the synthesis method. 

On the one hand, metal and metal-based compounds are commonly used to fabricate antimicrobial composite membranes involving copper. Among these metal-based compounds it is possible to mention metal-polymer complex and coordination polymers. The metal-polymer complexes can be obtained on the basis of heteroaromatic polymers, whose backbone was functionalized by units containing functional groups capable of forming coordination bonds with transition metals, particularly copper(I) or copper(II) salts [128]. In this way, the linkers anchored to polymer act as chelating arms to coordinate copper ions, promoting the metal-polymer complex formation. Thus, linkers with carboxylic, sulphur and amine groups are desirable. 

On the other hand, coordination polymers contain metal ions linked with coordinated organic ligands into an infinite array. Coordination bonds must define this infinite array [129]. These compounds have attracted attention because of the different architecture that can be formed and the several physicochemical properties that can be included in a modified membrane. The development of new systems based on copper is strongly studied due to the different characteristics of copper already mentioned. Copper is a versatile type of building block that has been successfully used for the synthesis of coordination polymers in combination with different neutral ligands that can offer appreciable properties. The selection of additives to form copper-based complex materials is very important, since the overall performance of the modified membranes rely only on it. Thus, the use of copper complexing and chelating agents mainly aims at stabilizing the copper on the membrane, controlling the copper ion dissolution and improving hydrophilicity of the membrane surface. 

Therefore, different copper-based materials (composites, metal-polymer complex, coordination polymers) have been synthesized to be incorporated into the membrane performance. Some examples reported are PANI-CuNPs, Chitosan-CuNPs, Cysteamine-CuNPs, CuBTTri-MOF, PEI-CuNPs, Cu-BTC-MOF, Cu^2+^-DOPA, DOPA-Cu^2+^/PEI-CuNPs, PDA-rGO-Cu, TA-Cu^2+^, CoFe_2_O_4_/CuO-NPs, Cu/TNTs, CuO/ZnO, Cu[DNDP]_3_MWCNT, Cu/Sepiolite, and Ag_3_PO_4_/ZnAlCu-NLDH. Overall, the notable use of copper materials such as metallic and oxide nanoparticles, salts, composites, metal-polymer complexes, coordination polymers for modifying MF, UF, NF, FO and RO membranes have been reported, and the analysis of the benefits and limitations of these proposals is presented in the following section.

## 3. Polymeric Membranes Modified by Copper Incorporation

### 3.1. Reverse Osmosis (RO) Membranes

Reverse osmosis desalination is the most widely used technology worldwide [14,21,22,130]. «In 2019, 21,123 desalination plants» worldwide were distributed in 170 countries, which supplied more than 100 million m^3^/day of fresh water to supply more than 300 million people [131]. This process implies the use of semipermeable membranes, where feed water is forced through the membrane when an external pressure force is applied, and salt ions and contaminants are rejected [132]. 

The most known polymer membranes have been made from polymers with aromatic polyamide groups such as thin-film composite (TFC), which dominate the RO membrane field nowadays given their great water flux and high solute rejection, but they are not completely resistant to fouling [14]. As it was mentioned above, membrane fouling is considered an inevitable obstacle affecting seawater desalination plants, causing a decrease in membrane performance [133]. As was previously mentioned, the physicochemical properties of membrane surface [21,23,127], such as hydrophilicity, roughness and electrostatic charge are the main factors influencing membrane fouling [17,108,117]. In order to enhance RO membrane properties, most research has involved the introduction of hydrophilic layer, the reduction of surface roughness, and the improvement of charge property to favor the electrostatic repulsive force between the charged membrane surface and the foulant [8,13,27,134]. 

Among the various proposals to modify RO membranes with anti-biofouling effect, there are a significant number of research related to copper-modified TFC membrane. In addition to the benefit of copper on the anti-biofouling effect of the membrane, given its toxicity, its impact on the surface properties of the material have also been analyzed. Different copper-based materials such as copper hydroxide nanoparticles, CuO-NPs, Cu-NPs, copper ions and copper-based MOFs have been used (Table 2). Moreover, different modification techniques to incorporate these copper materials into TFC-RO membranes have been proposed. These include a) the modification of the commercial membrane surface, through different methods such as coating, grafting and layer by layer assembly. They consider, in some cases, the use of stabilizing agents or linker agents in order to control the copper ion dissolution and the modification stability, and b) the immobilization of the modifiers fillers within polymeric matrix during the interfacial polymerization process (IPP), to modify the polyamide active layer [135]. In this review, the strategies addressed in these investigations will be widely discussed in terms of the influence of incorporated copper on the anti-biofouling effect, the surface properties, and membrane performance. In addition, the advantages and disadvantages presented by the employed modification techniques are discussed. All of these features are discussed with a view towards a possible industrial applicability.

As can be seen, the coating technique has been employed by several authors to modify TFC-RO membranes with copper-based materials. For instance, Kankanechi et al. reported the adsorption of copper hydroxide on TFC-RO commercial membranes by coat of membranes with copper hydroxide (Cu(OH)_2_) solution to produce anti-biofouling membrane. Hence, the anti-biofouling properties were attributed to the release of Cu^2+^ ion given the modified membrane. The authors demonstrated that this beneficial effect is dependent on the pH of the treated solution. Thus, the bacteria killing ratio increased when pH was increased from 6.5 to 7.5 [76]. Despite this significant improvement in the anti-biofouling effect, authors stated that the stability of this modified membrane was not adequate for long operating times due to loss of effectiveness. 

**Figure 3:**
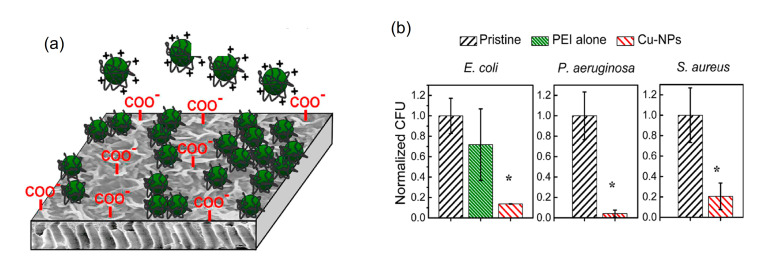
Results of the functionalization of TFC-RO membranes with Cu-NPs reported by Ben-Sasson et al. [32]. (**a**) Schematic of the electrostatic binding between the Cu-NPs (positively charged) and the carboxyl groups (negatively charged) on the active layer of the pristine membrane. (**b**) Biocide capacity comparison between the pristine membrane (black) and modified with the capping agent (PEI) alone (green) and Cu-NPs (red) over *E. coli*, *P. aeruginosa* (gram negative bacteria) and *S. Aureus* (gram positive bacteria). Asterisks (*) indicate a statistically significant difference between the functionalized and pristine membranes (*p* < 0.05). Adapted from [32].

Additionally, Ben-Sasson et al. reported the functionalization of TFC-RO membranes with Cu-NPs using two ways. The first way consisted in the dip-coating of the membrane surface with a previously synthesized Cu-NPs solution. These Cu-NPs were synthesized through a wet chemical reduction employing the polycation polyethylenimine (PEI) as capping agent, which imparted positive charge to the surface of the Cu-NPs which promoted the electrostatic interaction with the negative charge of membrane surface because of its native carboxylic groups (see Figure 3a). A remarkable anti-biofouling effect was observed with 80% to 95% anti-adhesion capacity for three model bacteria strains (*E. coli*, *P. aeruginosa*, and *S. aureus*) attributed to the toxicity of the bound Cu-NPs (Figure 3b). With this modification, the surface properties (hydrophilicity and roughness) of the modified membrane were not affected, and they also presented flux and rejection salts similar to the pristine membrane. However, this modification had a relatively rapid dissolution rate of the Cu-NPs, with dissolution of more than 30% of the loaded copper during the first two days, which could promote an early depletion of incorporated copper [32].

The second way was the coating of membrane surface through the in situ formation of Cu-NPs using sodium borohydride as a reducing agent without the use of a capping agent. Insignificant changes in the membrane surface charge and roughness after modification were observed. However, the surface of the modified membrane was slightly less hydrophilic, with contact angles increasing from 45.46 ± 1.68° to 59.84 ± 3.13° for the pristine and in situ modified membranes, respectively. A minor increase in the membrane water permeability coefficient and slightly decrease of salt rejection were observed on modified membranes [40]. Moreover, water physicochemical parameters and hydrodynamic conditions in desalination plants might accelerate the Cu-NP dissolution on these conditions.

Furthermore, the grafting approach has also been employed to incorporate Cu-NPs in TFC-RO membranes by in situ NPs synthesis in the presence of different linking agents such as polyaniline (PANI) [77], carboxylate chitosan (CCTS) [78] and cysteamine (Cys) and GO [79]. Thus, Khajouei et al. reported the grafting of PANI on TFC-RO membrane surface by in situ aniline polymerization under acidic conditions, and subsequently in situ Cu-NPs synthesis to produce a modified PANI-CuNPs-TFC membrane. The synergistic effect between PANI and Cu-NPs allowed obtaining a more hydrophilic membrane, with positive surface charge and slightly less roughness in comparison to the pristine membrane. The hydrophilic surface of the PANI-CuNPs-TFC membrane increased the water flux ~28% and salt rejection ~1% in contrast to the unmodified membrane. Anti-biofouling properties of the PANI-CuNPs-TFC modified membrane included high inhibition zone and long-term biofouling experiments showed constant and higher permeate fluxes than those shown by the unmodified membrane [77]. 

The hydrophilic natural polymers, such as CCTS, have been employed to coat the RO-TFC membrane surface. After treatment with CuCl_2_, aqueous solutions and cross-linking agent glutaraldehyde (GA) are used to reduce the CuNPs in situ and fix them in the cross-linked coating layer [78]. In this study, authors highlighted that it is very important to find an adequate concentration of polymer coating and crosslinking agent in order to not affect the membrane performance. These membranes showed excellent antibacterial properties with antibacterial efficiency above 99% after their immersion in deionized water for 90 days. These results suggested a long-lasting antibacterial performance of membranes, which was attributed to the slow release of copper ions, since the copper ions released from Cu-NPs can be absorbed by CCTS coating via chelating effect. Besides, these modified membranes showed better hydrophilicity, lower water flux, higher salt rejection and better protein fouling resistance. 

**Figure 4:**
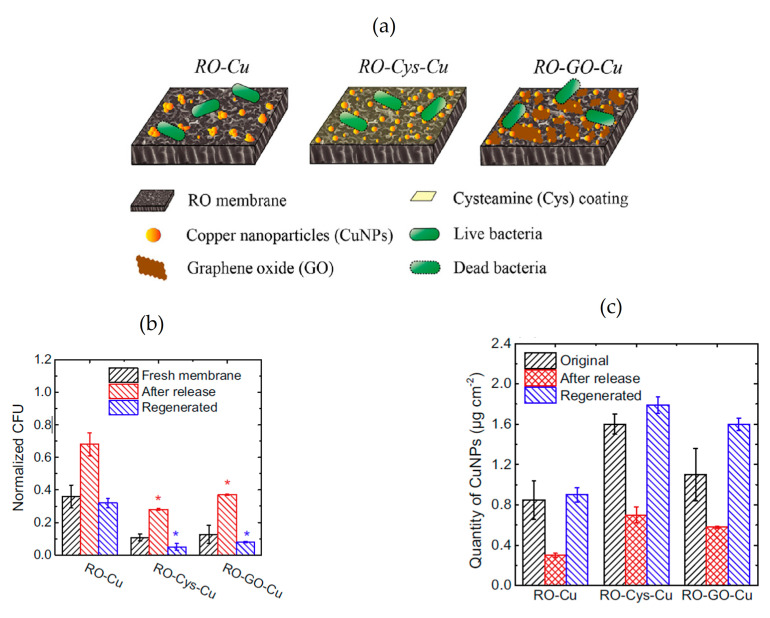
(**a**) Schematic of copper-modified RO membranes implemented by Ma et al. [79]. From left to right: Coating of membrane surface by in situ Cu-NPs reduction (RO-Cu), grafting of RO membrane with cysteamine linker and Cu-NPs (RO-Cys-Cu) and with graphene oxide linker and Cu-NPs (RO-GO-Cu); (**b**) Number of viable cells attached (CFU) in modified membranes compared to pristine membrane; (**c**) Quantity of NPs in each membrane after the release for a period of 7 days and after regeneration with Cu-NPs (note that the amount of NPs after regeneration is higher than the original amount of copper). Asterisks (*) indicate a statistically significant difference between the pristine and modified mem-branes (*p* < 0.05). All images and graphs are extracted from [79].

Additionally, Ma et al. conducted a comparative study in relation to the impact produced by the modification of RO-TFC membrane with Cu-NPs in two different ways. Namely, 1) the coating of membrane surface by in situ Cu-NPs reduction (RO-Cu), and 2) the grafting of RO membrane with Cys linker and Cu-NPs (RO-Cy-Cu), or GO linker and Cu-NPs (RO-GO-Cu) [79]. The study showed that the loading quantity of copper on a modified membrane surface can be improved using these linkers (see Figure 4a). This feature influences the anti-biofouling effect of membranes against *E. coli* bacteria that work as gram negative model bacteria, since the antibacterial and anti-adhesion effect decreased with depletion of the loading quantity of copper on membrane (RO-Cys-Cu > RO-Cu-GO > RO-Cu) (see Figure 4b). However, RO-Cys-Cu membrane was more hydrophobic while the GO linker produced more hydrophilic membrane. Authors concluded that the magnitude of the anti-biofouling effect is driven by the presence of copper in the membrane and the ability of the membrane to release these copper ions. Thus, the presence of Cys and GO linker induced a better control of the release behavior of CuNPs, promoting a gradual decrease of anti-biofouling effect until 7 days in a synthetic wastewater. Moreover, authors demonstrated the successful regeneration with Cu-NPs on the membrane surface after their depletion, evidencing the modified membranes’ potential for long-term application (Figure 4c).

From a slightly different point of view the incorporation of CuO-NPs [14], Cu-NPs [36], Cu-meta-phenylendiamine oligomers (Cu-mPD) [27], and water-stable Cu-based MOFs [80] into some monomer (m-phenylendiamine (MPD) or trimesoyl chloride (TMC)) during IPP has been explored as a promising approach to generate TFC-RO modified membrane, which in turn enhances anti-biofouling properties. 

Garcia et al. reported that the incorporation of CuO (1% wt) in MPD during IPP produced the CuO-TFC-RO modified membrane [14]. Unfavorable changes on the physicochemical properties of modified membrane surface were observed, i.e., similar contact angle, higher surface roughness and less negatively charged surface. In spite of this, the modified membrane showed anti-biofouling properties in the batch test, where *E. coli* acted as a model bacterium. Bactericidal effect and anti-adhesion ability were attributed to the release of copper ions from CuO-NPs, which have high ionic character, and the negative surface charge remained by the modified membrane, which underlines the anti-adhesion effect through electrostatic repulsion. An increase of water flux (~2 times respect to pristine membrane) was attributed to the hydrophilic character of CuO-NPs [14].

**Figure 5:**
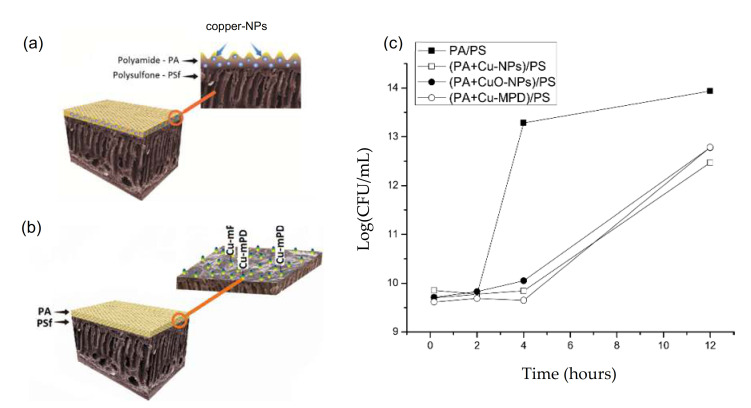
(**a**) Schematic of CuO-NPs addition to TFC-RO membranes during IPP and (**b**) of a TFC-RO membrane modified by formation of copper-oligomer complex (Cu-mPD) in situ. Extracted and adapted from [24]; (**c**) Bactericidal capacity (quantified by CFU) of copper modified membrane by addition in IPP of Cu-NPs, CuO-NPs and Cu-MPD compared to a pristine membrane (PA/PS). Extracted from [85].

A similar research reported the use of Cu-NPs (0.25%) during IPP to produce a Cu-TFC-RO modified membrane (see Figure 5a) [36]. This Cu-TFC-RO modified membrane showed excellent antibacterial effect (~99%) and good anti-adhesion effect (83%) in batch tests. The anti-biofouling effect was attributed to the higher reactivity of Cu-NPs provided by the release of toxic Cu^+2^ ions, in addition to the generation of ROS. Cu-NPs produce ROS in the medium that damages bacterial DNA causing bacterial death [136], and the release of ions increases intracellular ROS in the bacteria, thereby inflicting its death [137]. The modification produced a detrimental effect on desalination performance with decrease of water flux (~31%) and rejection salt (~2%) with respect to the pristine membrane, which was attributed to the increase of contact angle of modified membrane surface (>33% than pristine membrane) and the agglomerate formation.

The addition of copper chloride (1 wt%) in MPD monomers during IPP allowed the formation of Cu-mPD oligomer complex in TFC-RO membrane (Figure 5b) [27]. A mechanism for formation of the oligomer within the membrane was proposed based on the interaction between the oxygen of the carbonyl group of the polyamide layer and copper ion of the Cu-mPD oligomer complex. The modified membrane showed a slight decrease in hydrophilicity and higher surface roughness. However, this modified membrane showed excellent anti-biofouling properties with bactericidal and anti-adhesion effect >99% without compromising the membrane performance, with increase of flux water (~33%) and similar rejection salts with respect to the membrane pristine. 

Thus, the aforementioned studies suggest that there is a direct relationship between the type of copper material incorporated during IPP and the anti-biofouling properties of modified membranes with this approach. The bactericidal effect increases with the copper types incorporated according to the following sequences: Cu-mPD ≥ Cu-NPs >> CuO-NPs (Figure 5c). Differences in the dissolution level of copper-based NPs in membrane were noted, suggesting a dual-type effect that defined the copper toxicity into the membrane, associated to the dissolution capacity, which depends on the interactions between the copper and the polyamide (PA) layer of the membrane, and ROS production, which vary depending on the copper type [85].

Recently, Wen et al. explored the impact of the incorporation of water-stable Cu-based MOFs (CuBTTri) into the PA layer on anti-biofouling properties and desalination performance [80]. CuBTTri was incorporated in trimesoyl chloride (TMC) monomers during IPP reaction. The water flux decreased by ~70% in biofouling continuous flow test after 24 h with respect to the pristine membrane, which makes this fact dependent on MOFs dosages. This modified membrane showed a much lower Cu release rate than that in other studies using metal nanoparticles for the modification of TFC membranes (0.010 ± 0.001 µg/(cm^2^ day) after 3 days soaking) [87,138]. Therefore, the authors concluded that the release of Cu from the water-stable MOFs was not the dominant factor contributing to the antibacterial behavior in the long-term. Thus, the anti-biofouling test and the anti-biofouling behavior were attributed to the direct contact of bacteria with MOFs, which might cause the oxidation of functional groups of the bacteria, e.g., thiols [139,140], causing damage to the bacterial cells.

**Figure 6:**
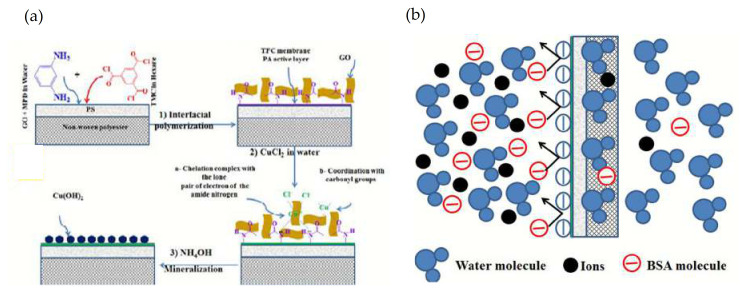
(**a**) Schematic representation of the IPP and mineralization of TFC-RO membrane with formation of Cu(OH)_2_ surface modification; (**b**) Mechanism of electrostatic repulsion between BSA and membrane surface. Extracted and adapted from [141].

The combination of two approaches (IPP and coating) has also been used to modify a TFC-RO membrane with enhanced anti-fouling properties [141]. Thus, the incorporation of GO during IPP and after the coating of this membrane with CuCl_2_ produced a TFC-Cu-GO membrane. An additional treatment of this membrane with ammonium hydroxide produced the membrane mineralization (M-TFC) by generation of Cu(OH)_2_ on the membrane surface (see Figure 6a). The mineralized membrane with optimum concentrations of copper showed higher pure water permeability and solute water flux compared to the pristine TFC membrane with an excellent salt rejection. Moreover, the antifouling tests using bovine serum albumin (BSA) as an organic fouling showed that TFC-Cu-GO and M-TFC membranes had an excellent antifouling property. This is due to the fact that copper hydroxide increases the hydrophilicity and the negative charge density of the membrane surface. The membrane hydrophilicity increased the water molecules onto the membrane surface and hence lowered the adhesion property between the foulant molecules and the membrane surface. In addition, the electrostatic repulsion between the membrane surface and foulant molecules increased due to the increasing negative surface charge density (see Figure 6b).

Finally, other approaches to modify commercial TFC-RO membranes have been scarcely reported. For example, the physical vapor deposition (PVD) approach has been reported to coat polyamide membranes with CuO-NPs employing metal-gas plasma, under optimal conditions of time plasma treatment, and current research obtained membranes with bactericidal activity to *E. coli* of 99%. Nevertheless, the materials were not tested under real operating conditions [86]. In addition, spray- and spin-assisted layer-by-layer self-assembly (SSLbL) method was reported to incorporate Cu-NPs in polyamide layers of RO-TFC commercial membranes [87]. Layer by layer (LbL) assembly was achieved by electrostatic interaction. The first layer was produced by interaction between the negative charge of polyamide layer and Cu-NPs stabilized with PEI (PEI-CuNPs), and subsequent layers were formed between poly(acrylic) acid (PAA) and PEI-CuNPs [87]. The spray and spin technique allowed to obtain uniform bilayer PEI-CuNPs/PAA on RO-TFC membrane in short times, increasing the number of bilayers that increased the copper load on the membrane. The modified membrane showed similar negative surface charge, slight increase in roughness and decrease in the surface hydrophilicity, which was attributed to the Cu-NPs loading. A ten-bilayer coating of the membrane resulted in only 13.3% reduction in the water permeation flux. Regarding anti-biofouling properties, the PEI-CuNPs/PAA modified membrane showed high antibacterial activity in the range of 94.3% to nearly 100%. Continued biofouling test showed 43% reduction of permeate flux for the PEI-CuNPs/PAA modified membrane, which was very close to the control LB solution without bacteria, showing 38% reduction of the permeate flux.

In summary, different copper-based materials (copper hydroxide, copper ions, copper oxide and copper metal nanoparticles and copper-based MOFs) have been used to modify TFC-RO membranes, mainly to promote the anti-biofouling effect. Although the mechanism of toxicity can be dependent on the type of copper incorporated, an impact on the effectiveness of this effect and the performance of the membrane is undoubtedly dependent on the method of modification. Different approach techniques such as coating, grafting, layer by layer assembly and interfacial polymerization processes have promoted excellent anti-biofouling effects. However, disadvantages such as the stability of modification, loss of effectiveness over time, limitations in long-lasting operating times are associated with the method. Coating/dip-coating is the most practical approach technique, but low stability of modified membrane limits its application during long operating times due to the loss of effectiveness by early depletion of incorporated copper. A strategy could be the incorporation of copper nanoparticles stabilized by stabilizing agents with nitrogenous functional groups in order to control the capacity of copper ion dissolution from the nanoparticle. In contrast, the grafting approach has allowed to obtain modified membranes with long-lasting antibacterial performance due to the slow release of copper ions given the chelating effect of different linker agents on copper ions. Nevertheless, membrane flux should be affected, and an adequate concentration of linker agent is required. In this context, systematic grafting studies with linkers that have high capacity to chelate copper ions and that can improve the water flux without blocking the porous should be addressed. Linkers such as branched polymers and graphene oxide with amine and sulfur functional groups are promising targets in order to achieve the aforementioned conditions. Finally, the immobilization of copper materials within a polymer matrix through their incorporation during interfacial polymerization process is another strategy. This promising approach allows to modify the membrane from the manufacturing process itself, which would enable tuning the properties of the new filter. However, several synthesis parameters should be optimized in order to avoid surface defects on the PA layer and the copper nanoparticles agglomeration, which impacts the membrane performance. Hence, this approach has great potential to produce copper modified TFC-RO membranes with tunable properties, but it might also require a long time to achieve its industrial implementation.

### 3.2. Forward Osmosis (FO) Membranes

Forward osmosis is an emerging technology primarily studied for water desalination with the advantage of offering an alternative way to purify saline sources using low-cost energy. Compared with RO technologies, the market relevance and science impact of FO is relatively small, but in the past few years there has been an increment in the amount of studies for this alternative membrane technology. This trend shows the efforts of the scientific community to diversify the conventional methods of purification of saline water sources using low impact methods in environmental, energy and economic terms [142].

Technically, the operation of FO is based on a dense hydrophilic semipermeable membrane that separates two sources with different concentrations called feed solution (FS) and draw solution (DS). The osmotic pressure gradient between them is the driving force process [143]. As well as RO, FO membranes consist of an active layer, which in theory possesses a high-water permeability, a low reverse solute permeation, and a support layer with high water mass transfer and high resistance to concentration polarization phenomena. However, these basic qualities should be accompanied by good antifouling properties, chemical resistance, and mechanical stability. Of course, finding materials with all these properties could be challenging. For this reason, some active layer materials used in RO or NF technologies have been implemented in FO studies, especially in desalination studies, that include multivalent saline feed treatments, waste-water treatments with large organic molecules and ionic contaminants. 

Among the typical RO active layer materials, the current research about FO materials points at cellulosic derivatives, polyamides and polyelectrolytes [143,144]. However, there are some limitations in relation to an overall low flux, principally due to unfavorable material characteristics, such as thick sponge-like substrates and compact supports. These largely block the water mass transfer and cause a high internal concentration polarization (ICP) inside the support. The ICP is an important challenge for the performance of FO processes, and is influenced by some membrane sublayer characteristics including porosity, tortuosity, and thickness. The structural parameters (S, thickness and X, tortuosity/porosity) are a strong indicative value in order to quantify the structural contribution of the FO membrane in the ICP effect [143,145]. In this sense, the modification of FO membranes has been mainly directed to the increase of water mass transfer, structural membrane parameters and avoiding the ICP phenomena. Studies conducted on the modification of membranes with copper materials have thus aimed at the already mentioned benefits and have also targeted antibacterial properties (Table 3). The main studies on copper modified FO membranes are related to the introduction of Cu-based MOFs as load removable filler to prepare MOFs-based polymeric substrates through a phase inversion method, or the modification of the active layer by this kind of particles using in situ polymerization techniques. Hence, the approaches of these studies will be described in relation to the effectiveness of the copper modification regarding the properties achieved in terms of water mass transfer, antifouling effects and ICP resistance. 

For instance, Lee et al. [72] explored the use of metal organic frameworks: copper based Cu-BTC MOF (HKUST-1 or copper benzene-1,3,5-tricarboxylate) were used as a removable filler for preparing MOFs-based porous membranes by phase inversion method using a polymeric dope solution of polyacrylonitrile (PAN). The resulting substrate seemed to have an improved water mass transfer and ICP controller support for the active layer synthesized later by LbL conventional technique, leading to the final FO membranes. In the case of the copper MOF, the overall membrane bulk porosity (porosity 85%) and the hydrophilicity (contact angle 44° to 29°), compared to the original PAN, increases, resulting in a reduced structural parameter from 360 µm to 190 µm, high water flux (>130 L m^2^ h) and a decrease of the material tortuosity, mediated through the macropore formation in the support. 

**Figure 7:**
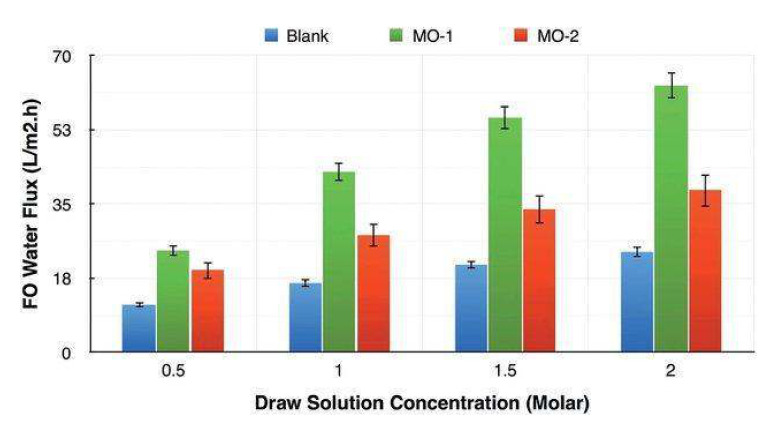
FO water flux of the membranes in atomic layer deposition (ALDS) mode and using different DS concentrations (T ¼ 25 C, feed ¼ DI water), error bars represent standard deviation over runs. Extracted from [73].

On another note, Zirehpour et al. [73] studied the effect of the addition of the same Cu-BTC MOF to improve the performance of cellulosic based FO membranes with the aim of increasing the overall porosity, pore interconnectivity and membrane hydrophilicity. These FO modified membranes were prepared by phase inversion via the immersion precipitation method using a dope solution of cellulose acetate/triacetate (CA/CTA) mix loaded with a casting MOF solution. The results show a significant reduction in the structural parameter of the modified polymeric matrix from 579 µm in the original CA/CTA matrix to 136 µm in the cellulosic MOF based membrane. The water permeability increased by 72% and the water flux was enhanced about 180% with respect to the polymeric unmodified matrix (see Figure 7). The overall porosity increased in form less than 70% to almost 90% followed by an increase in the hydrophilicity, all compared with the unmodified cellulose matrix. These combined effects enhanced the mass transfer of water and seem to control the effect of ICP in the membrane during the osmotically driven process.

One more similar report from Dai et al. [74] used the Cu-BTC synthesized as layered 2D particles to modify the PA active layer of a TFC-FO membrane using interfacial polymerization method. In this case, the reported TFC-FO exhibited an increment of 50% in the water flux with respect to the unmodified membrane in membranes, loaded with 0.12% of Cu-BTS layered particles. The membrane showed an important increase in hydrophilicity and a decrease in the structural parameter, from 402 µm to 366 µm. The authors highlighted the theoretical potential of antifouling and antibacterial effect without any directed test but provided by the micrographic presence verification of these Cu-layered particles in the obtained surface.

**Figure 8:**
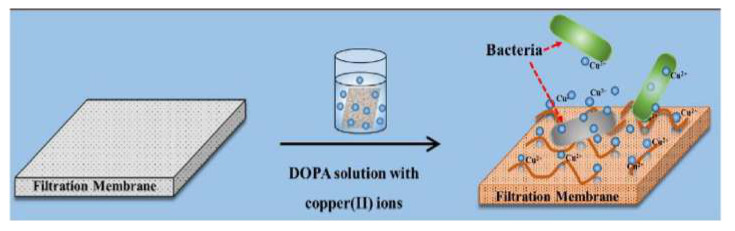
Schematic of the one-pot chelating copper ions modification on FO membrane. Copper ion release and interactions between bacteria and the copper-containing surface results add biocide properties. Extracted from [75].

Only one recent study reported by Liu et al. [75] involved the modification of the active layer of an FO polyamide membrane with a copper-dopamine complex using the one-pot chelating Mussel-inspired method. The aim of this work was to generate a cost-effective method for mitigating the effect of biofouling on various surfaces, including the FO membranes. The oxygenation during the reaction stage showed an accelerated DOPA polymerization with high incorporation of copper cations in the active layer (see Figure 8). The membranes showed good antibacterial properties against *S. aureus*. Despite the antibacterial success, the membranes exhibited a comparative decrease of water flux of 28%, attributed to a blocked surface effect, with no reported structural parameters. 

In summary, modification of FO membranes by copper incorporation has been directed mainly to the increase of water mass transfer, structural membrane parameters and avoiding the ICP phenomena. For this purpose, MOF-based polymeric substrates can be obtained using different strategies. For example, the phase inversion method or the modification of the active layer by this kind of particles using in situ polymerization techniques. Despite the good results regarding the structural parameters and the water mass transfer using proper amounts of particles load, a high loading percentage could lead to severe particle agglomeration in the membrane support/active layer, and subsequently may have an adverse effect on the membrane properties and performance. The best results in all studies were given with exceptionally low copper MOF particle loads. However, the high solubility in water of these particles can lead to serious losses of the properties acquired in short times, due to the low concentrations. This might affect the levels of porosity and tortuosity due to dissolution phenomena during the FO, and consequently the geometrical dependent S parameter. Conversely, copper-dopamine complex by one-pot chelating Mussel-inspired method is another alternative route for the modification of the active layer of an FO polyamide membrane. Given the simplicity of this approach, this technique could be important in further investigations for treating biofouling problems but should be adjusted to acceptable parameters for a performance on osmotically driven processes.

### 3.3. Nanofiltration (NF) Membranes

The implementation of nanofiltration processes started three decades ago with a promising membrane-based separation method directed to give a solution to the problem in different areas, such as drinking water, wastewater treatment, partial desalination, chemical separation, and other industries. In fact, the NF process provides an intermediate approach between UF and RO processes, rejecting particles of about 1 nm, which corresponds to a minimal molecular weight cut-off (MWCO) of 300 Da (including oligomers, polyvalent ions, and similar species), pressure operation between 3–20 bar, pore size range between 0.5–2 nm and permeabilities around 1.5–30 L/(h m^2^ bar^−1^).

The challenges of all pressure driven technologies in NF are strongly linked with membrane fouling/biofouling, insufficient separation, generation of concentrates, membranes short lifespan, low chemical resistance and deficient rejection for particular compounds [146]. Regarding the separation, it is well known that NF membranes typically had low rejection of monovalent ions, high rejection of divalent species and higher fluxes at low pressure than RO techniques. This specificity allows the NF technologies to be used in applications that require specific separations in wastewater treatment and fine-chemistry areas especially, such as within the pharmaceutical and biotechnology fields, where the removal or concentration of a determined compound is required. The mentioned technical development allows the NF to be used in multiple specific applications in diverse industries, with well-established membrane, modular and processing development [147].

Conversely, there are still several active trending investigation lines especially environmental applications, membrane fabrication/modification, fouling, desalination, dye wastewater treatment and process modeling [147,148,149]. Regarding the water treatment area, the wastewater treatment (WWT) is undoubtedly the most common probed application of NF with around 18% of the studies in the past decade, combined with water-related investigation in fouling, desalination and membrane design topics [17,148,149]. While studies in WWT are generally concerned with the removal of specific molecules or ions, studies in desalination generally focus on pre-treatment or partial desalting. Membrane design topics include the development of advanced techniques for membrane preparation using different strategies such as blending, interfacial polymerization, layer by layer, grafting, nanoparticle incorporation or beam irradiation, and reporting enhancement of properties such as antifouling, antibiofouling, hardness removal, dye removal and dissolved salts removal [150]. 

In this connection, the antimicrobial activity is an appreciated enhancement due to costly biofouling problems in NF. An excellent alternative is the use of copper-based modifications that, in the case of NF technologies, had a limited but promissory number of related studies. Thus, the research reported to date combining nanofiltration and the use of copper as a modifier is divided in WWT composite membranes and pretreatment desalination composite membranes, prepared usually by blending/phase inversion techniques combined with a pool of complex advanced methodologies illustrated in Table 4. Some of them objectively stated the bactericidal enhancements of copper-based modifications, while others were directed to the improvements of hydrophilic properties and surface/pore characteristics. In these regards, the studies will be analyzed based on the preparation strategies and success of modifications regarding the rejection of species, water flux, hydrophilicity, membrane fouling effect and antibacterial activity. These, depending on the case, can be applied for two main applications: wastewater treatment and desalination. 

First, the studies including copper-containing membrane preparation and design for WWT are dominated by the PAN matrix alongside with one research output with a CA based membrane. In the latter case, Asapu et al. [63] developed a low-biofouling membrane mediated by de CA functionalization with glycidyl methacrylate (GMA) combined with iminodiacetic acid (IDA) as chelating agent for copper divalent ion fixing. The methodology involves a homopolymerization and a phase inversion stage followed by an immobilized metal affinity (IMA) based chemistry reactions. The authors propose the development of a simple method using readily available and cost-effective materials such as cellulose-based polymers, thus fixing an antibacterial agent only by coordination interactions. The antibiofouling properties show a biocidal effect against *P. fluorescence,* manifested in the reduction of biofilm area formation and a lower flux rate decline compared with unmodified membranes, after organic foulant filtration essays (BSA, Lipase). These results suggest an increase in lifespan and antibiofouling/antifouling properties for the new membranes. However, the rejection rates on salts and organic contaminants are not reported to hinder the objective analysis of the NF membrane efficiency. In addition, the cooper leached from the membranes seems to be low, confirming the stability of the modification.

**Figure 9:**
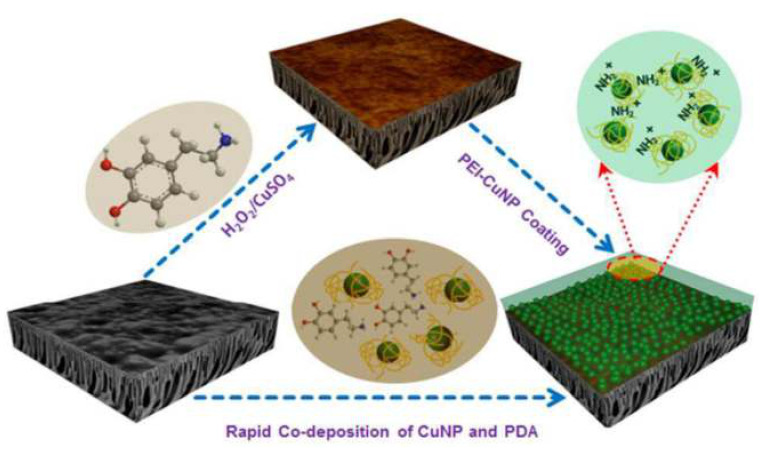
Schematic diagram of the surface modifications of the HPAN membrane via two-step deposition and co-deposition using PDA and CuNPs. Extracted from [64].

The PAN-based studies show similar behaviors despite the big differences among modifications. For example, Zhu et al. [64] developed two modification strategies using two step deposition and co-deposition employing Mussel-inspired polydopamine (PDA) chemistry for the immobilization of polyethyleneimine-functionalized copper nanoparticles (PEI-CuNPs) on a PAN-UF template, generating a loose NF membrane (see Figure 9). The treatment reduced the pore size of the membrane by bridging the pore cavities. The results of the modification showed an enhancement of hydrophilicity, homogeneous particle distribution and low roughness. Regarding the operational parameters, the modified membranes show low salt rejection in both divalent and monovalent salts, and superior rejection against different textile dyes (0.6–2 kDa), offering the possibility to be used as dye concentrator in textile WWT. The antibacterial activity was extremely high against *E. coli,* with 93.7% of inhibition. 

Another similar work, by Zhu et al. [42], describes the nanocomposite membrane preparation of an NF material using reduced graphene oxide-copper (rGOC) nanocomposites to bridge the pore cavities of a PAN hydrolyzed membrane by a Mussel-inspired PDA modification. The obtained membranes had high water permeability, hydrophilicity, and strong antibacterial performance (97% against *E. coli*). In addition, the salt rejection was low for divalent and monovalent salt and the rejection against dyes was remarkably high (99.4%), confirming a potential use as a fractionator of textile dyes in WWT. 

Finally, another similar research by Chakrabarty et al. [65] used a simple tannic acid-cupric acetate complex (TA-Cu^2+^) functionalization to coat porous PAN hydrolyzed UF membranes through surficial co-deposition with gallol (1,2,3-trihydroxyphenyl) and catechol (1,2-dihydroxyphenyl) bio-polyphenols via polymerization. The structural analysis confirms the formation of a thin layer with good hydrophilicity properties and high pure water flux. Once again, the rejection of divalent and monovalent ions is low, while the filtration percentage of textile dyes and polymer molecules is remarkably high (99% 0.6 kDa), showing a membrane with potential use in desalting of organic solutes or WWT in contrast with the use of small organic molecules. Although those works are particularly directed towards the treatment of dyes or organic molecules, a low rejection on divalent salt has been observed. There is a detrimental influence given the modifications on the reduction of the pore size of the original UF membranes to the NF range, either due to inhomogeneities in the surface modification or low functionalization rates

Second, four reports on desalination applications for copper-modified NF membranes contain sulfonated polymers that work as the main starting point: polysulfone (PSf) and polyethersulfone (PES). The remaining two studies are related to the modification of alternative NF matrixes as PAN and PEI. In almost all these cases, the preparation of the membrane and its final structure behave as typical developments in reverse osmosis: a porous supporting polymer base covered by a dense active thin layer. 

For instance, Isloor et al. [66] describe the membrane surface modification of a PSf/polyisobutylene-alt-maleic anhydride (PSf-PIAM) matrix through direct PVD of elemental copper. The resulting coating is reported to be highly homogeneous for its application in desalination and antibiofouling purposes. The pure water flux is about 36 $L/h\;m^{2},$ with a monovalent salt rejection of 96% (3500 ppm NaCl), proving its desalination capacity. The antimicrobial effect was probed against *B. cereus* through qualitative inhibition halo formation. The less overall flux decline of the membrane suggests a successful antibiofouling property even with the slight increment of the hydrophobicity of the phase inversion in the obtained membrane. In this case, the main concern with respect to this methodology is the release of metallic copper exposed to oxidation-reduction processes in an aggressive saline environment, which can lead to a rapid leaching of the deposited layer by physical methods, and a final short membrane lifespan.

On the other side, Misdan et al. [67] made a simple chemical modification loading different concentration of copper benzene-1,3,5-tricarboxylate (CuBTC) nanoparticles on a PSf matrix by blending, followed by and interfacial polymerization to form a poly (piperazineamide) (poly-PIP) selective layer. The modification of the substrate support altered the thin layer properties, increasing the hydrophilicity and promoting layer densification. The results show a clear enhancement of the rejection performance especially for divalent ions (97% MgSO_4_) and organic foulants (99.9% BSA). Despite the good performance results, no leaching study is observed to verify the stability of a highly soluble species such as copper MOF, since its presence in the support membrane (permeate side) could generate the release of copper ions in the desalted water.

In the same line, another study related to PSf-(poly-PIP) based matrixes is described by Tajuddin et al. [68]. This modification consists in thin film composites fabrication by incorporation of copper-aluminum layered double hydroxide nanofillers (Cu-Al LDH), directly in the interfacial polymerization step. After the incorporation of layer double hydroxide (LDH)-type nanostructures, the morphology of the membrane surface was smoothened and its nodular characteristics decreased, also its hydrophilicity increased. Regarding the desalination performance, the results show an outstanding divalent ion rejection (MgSO_4_ 95.4%, Na_2_SO_4_ 96.8%, MgCl_2_ 95.6%) and partial desalination performance against monovalent ions (NaCl 60.6%). In addition, the pure water flux was increased by a significant amount (15%), and the enhancement of antifouling properties was probed against an organic foulant (cetyltrimethylammonium bromide, CTAB). However, despite the good structural and morphological characterization exposed, there is an absence of necessary characterizations to verify the stability of the LDH nano-filler compounds, which are chemically sensitive to dissolution in aqueous medium. This phenomenon could generate the presence of copper and aluminum ions in the resulting permeate, and/or defects in the integrity of the active layer of the NF membrane, compromising the effectiveness of the modification.

In another study, Zareei et al. [69] prepared a composited PES-based membrane loaded with CoFe_2_O_4_/CuO nanoparticles using blending followed by phase inversion technique. The resulting matrix exhibited higher hydrophilicity and low roughness. The performance parameters, such as pure water flux and salt rejection, showed a remarkable increase (95% Na_2_SO_4_, 72% NaCl); the rejection of heavy metal proved to be successful with more than 85% rejection of Cu^2+^, Ni^2+^and Pb^2+^. In this case, the asymmetrical membrane exhibited high void formation and bigger channeling in the polymer matrix with the NPs load. Surprisingly, these pore irregularities do not show adverse effects on the membrane performance, on the contrary, the desalination properties were critically enhanced. An antibiofouling study should be necessary to take advantage of the high concentration of loaded copper. Nevertheless, again, it is necessary to address quantitative studies of copper lixiviation stability to avoid Cu ion liberation on the wrong side of the filtration process.

Alternatively, Sumisha et al. [70] used PEI for the fabrication of NF membranes functionalized with TiO_2_ nanotubes and hydrogen trititanate (H_2_Ti_3_O_7_) nanotubes (TNT), by means of simple dispersion and phase inversion methods. One of the modifications includes the functionalization with TNT-based nanotubes, previously charged with copper salts via ion exchange doping treatments (Cu-TNT). The resulting mixed matrix membrane showed a macro-void pattern morphology. An enhancement in hydrophilicity was reached directly related with Cu-TNT functionalization. The salt rejection against divalent ions was acceptable in terms of softening process (80% K_2_SO_4_-45% CaCl_2_), but in general lines is lower than the conventional NF membranes. The monovalent ion rejection is surprisingly high regarding the macro-void morphology (75–65% NaCl), but only at lower salt concentrations (500–2000 ppm). The performance in pure water flux reached the maximum in the comparative study with the addition of Cu-TNT, also with good fouling properties against protein absorption. Nonetheless, despite the novelty of the modification, mainly by the use of alternative components, the rejection and recovery rates are not adequate for desalination, even against divalent ions. For instance, the water softening and brackish water treatments could be a feasible application.

**Figure 10:**
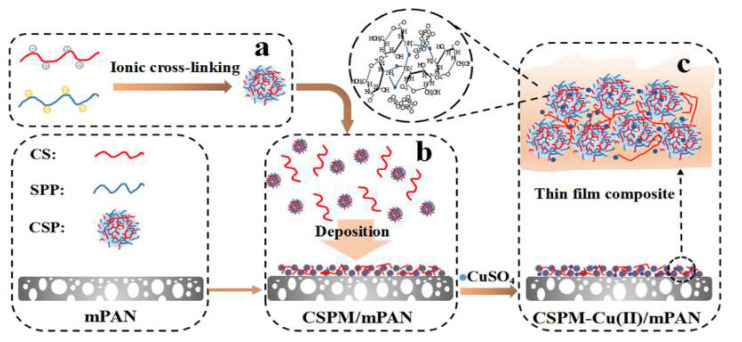
Diagrammatic sketch of membrane fabrication process of CSPM-Cu(II)/mPAN: (**a**) formation of CSPs, (**b**) deposition of CSP suspension onto mPAN to form CSPM/mPAN, (**c**) Cu(II) chelation of CSPM/mPAN to form CSPM-Cu(II)/mPAN. Extracted from [71].

Finally, Zhao et al. [71] describe the deposition of an active chitosan (CS)/polyphosphate (SPP) active layer onto a hydrolyzed PAN support by means of ionic crosslinking and LbL deposition, followed by a functionalization with copper divalent ions (see Figure 10). The resulting PAN-CS-SSP-Cu thin film composite membrane showed better hydrophilicity and higher pure water flux combined with a notable increase in salt and organic molecule rejection. In addition to this, the modified NF membrane exhibited a total antibacterial effect against *E. coli.* The main risk for this membrane is the long-term stability due to Cu(II) leaching and CS high rate degradation. However, the described method is an interesting approach for incorporating another more stable polyelectrolyte in high performance substrates.

In summary, copper modified NF membranes for WWT and pretreatment desalination have been prepared mainly by blending/phase inversion techniques combined with a pool of complex advanced methodologies. The anti-bactericidal effect and the improvements of hydrophilic properties and surface/pore characteristics have been the main focus of the modification. Regarding WWT, the studies are dominated by the PAN matrix alongside with one research output with a CA based membrane. Although those works are particularly directed towards the treatment of textile dyes or oligomeric organic molecules, the general divalent salt rejection is surprisingly low. These results suggest that modifications may have some problems in reducing the size of the original UF membranes to the NF range, either due to inhomogeneities in the surface modification or low functionalization rates. Regarding the desalination applications, copper-modified NF membranes based on sulfonated polymers such as PSf, PES and PAN modification, have enhanced the rejection performance. Moreover, water flux and salt rejection showed a remarkable increase, however, salt rejection is still lower than RO. Considering some factors such as low pressures and high fluxes, the modified NF approaches could be an excellent pretreatment complement in the water desalination fight against fouling/biofouling.

### 3.4. Ultrafiltration (UF) Membranes

Ultrafiltration is a membrane filtration process similar to reverse osmosis, using hydrostatic pressure to force water through a semipermeable membrane, with several operational advantages, such as low operating pressures, environmental operating temperature and low operating cost. Ultrafiltration membranes generally have pore sizes between 1 and 100 nm (Figure 1), making them an attractive option for the separation and removal of particles, bacteria and viruses from different water sources. Thus, this technology is used in a wide range of applications, such as RO pretreatment, production of drinking water and wastewater treatment. 

UF membranes are typically made of polymeric materials such as PES, poly (vinylidene fluoride) (PVDF), PEI, PAN, PSf, among others. In this regard, PES can distinguish it from others considering its suitable chemical properties, thermal stability, and appropriate resistance to the oxidation process. However, its inherent hydrophobic property often causes significant membrane fouling due to the adsorption of nonpolar solutes, hydrophobic particles and bacteria, affecting widespread applications of membrane processes. Therefore, various efforts have focused on preventing fouling, biofouling, and increasing membrane flux properties in this type membranes [151,152,153,154,155]. Some research has been carried out on the surface modification of UF membranes through the introduction of hydrophilic polymers and inorganic particles. To date, various modification methods, including chemical grafting [156], surface coating [157] and blending-crosslinking [158], have been employed to improve the hydrophilicity and the antifouling properties of these membranes. 

In this context, modifications of UF membranes through the incorporation of copper-based materials, mainly as nanoparticles, have been carried out in order to improve these properties. A comparative summary of the modifications studied by the different authors is shown in Table 5. In the analysis the following is considered: the different base polymers used in the production of modified UF membrane (PES, PVDF, PEI, PAN, PSf, among others), the different copper- based materials proposed for the modification and their impact in the properties mentioned before.

Studies on modifications of UF membranes based on the PES-based polymer matrix have generated promising expectations in obtaining membranes modified by the incorporation of copper-based nanoparticles (Cu-NPs, CuO-NPs, Cu_2_O-NPs) or copper-based composites through the phase inversion process, in order to improve antifouling, anti-biofouling properties and filtration performance. For instance, the incorporation of Cu-NPs synthesized into PES membranes in the phase inversion process have been studied by Akar et al. and Zhang et al. Both studies evaluate the permeability and the anti-fouling performance in the rejection of BSA [49,55]. They found improvements in the hydrophilicity, the permeability performance, protein rejection ratio and antifouling effect on copper-modified membrane with respect to the neat membrane. These results were associated with hydrophilicity of Cu-NPs and their capacity to migrate to the membrane surface during the phase inversion process in water, making the membrane surface more hydrophilic. 

**Figure 11:**
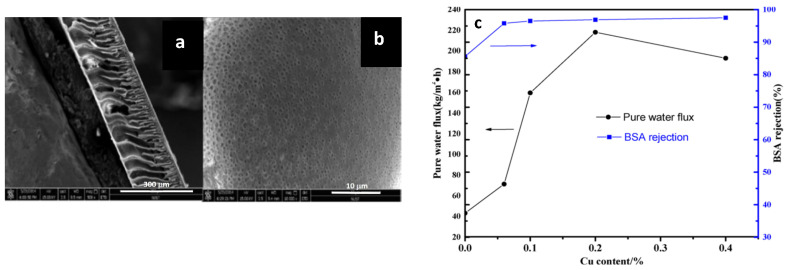
PES UF membranes modified by the SPAES and Cu-NPs incorporation. SEM images of PES/SPAES (**a**) and PES/SPAES/Cu-NPs membranes (**b**). PWF and BSA rejection of the PES/SPAES/Cu NPs membrane (**c**). FRR of the PES/SPAES/Cu-NPs membranes to humic acid (HA), sodium alginate (SA) and BSA. Adapted from Zhang et al. (2018) [55].

For instance, Zhang et al. showed that modified membranes exhibited an increase in porosity along with decrease in pore size by the incorporation of Cu-NPs (Figure 11a,b). In addition, the use of sulfonated poly (aryl ether sulfone) (SPAES) as an additive of casting solution in the presence of Cu-NPs produced modified membranes with highest rejection of BSA and flux recovery ratio (FRR) [55]. Thus, the introduction of sulfonic acid derivatives from SPAES and Cu-NPS within the casting solution could promote a synergistic effect, through the orientation of the hydrophilic group and the migration of Cu-NPs towards the membrane surface, favoring the increase of membrane hydrophilicity. Moreover, the presence of Cu-NPs and SPAES increases the viscosity of the casting solution. This could lead to a slower diffusion of the non-solvent and a delayed phase separation during the immersion precipitation process, resulting in the decrease of porous density. Consequently, the membranes with high Cu-NPs content showed high BSA rejection, but the pure water flux (PWF) was affected detrimentally (Figure 11c). Thus, the incorporation of additives into the casting solution, such as polymers with hydrophilic functional groups, could generate channels that promote the NPs migration to membrane surface and increase the membrane hydrophilicity, although low Cu-NPs content should be considered. This could be used as a strategy for the incorporation of Cu-NPs in PES-based UF membranes. 

**Figure 12:**
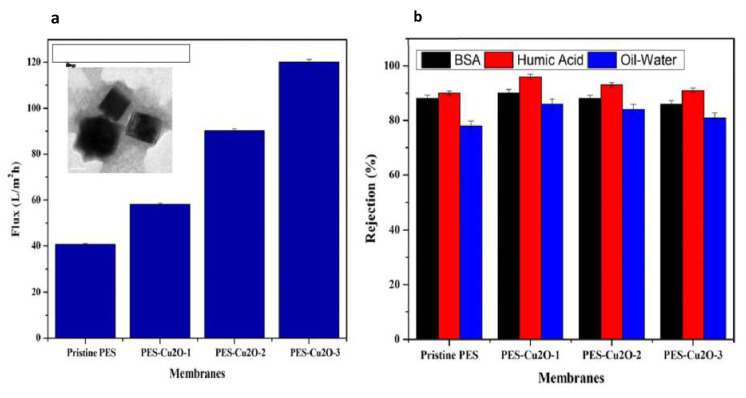
PES UF Membranes performance. PWF of neat membrane with respect to modified membranes given their incorporation into Cu_2_O-NPs, the inset figure exhibits a TEM image of Cu_2_O-NPs (**a**) and rejection of BSA, HA and Oil-Water (**b**). Modified from Pravallika et al. (2016) [50].

Studies of incorporation of CuO-NPs in UF membranes based on PES using the phase inversion method have mainly focused on the impact of the size and morphology of CuO-NPs on the hydrophilicity, permeability and antifouling properties of the modified membranes. For instance, Nasrollahi et al. and Pravallika et al. incorporated CuO-NPs with plate form, length sizes about 500–600 nm and thickness about 60 nm and cubic Cu_2_O-NPs with a size of around 28 nm in PES membranes through a phase inversion process, respectively [50,61]. A decrease in the contact angle and increase of the water flux on the modified membrane relative to the PES bare membrane were observed. Furthermore, the antifouling performances and the FRR (%) were also evaluated with different feed solutions: BSA, humic acid (HA), and oil–water (O/W). All modified membranes exhibited better antifoulant properties and FRR (%) relative to the pristine PES membrane (Figure 12). Similar to Cu-NPs, the hydrophilic properties of the modified membranes are related to the mobility of the CuO-NPs towards the membrane surface during the phase inversion process, due to the CuO-NPs high affinity for water. 

Another CuO-NPs inclusion study was developed by Nasrollahi et al. They focused on the prior functionalization of CuO-NPs with the amine 3-(aminopropyl) trimethoxysilane for incorporation into PES membranes [62]. Amine functionalization was used to improve the adaptability of the surface of these nanoparticles (hydrophilic) with a polymer surface (hydrophobic), as well as to increase the stability of dispersion in the media. Therefore, a decrease in the contact angle values, increase in the PWF and antifouling effect with 98% BSA rejection in comparison to the bare PES membrane were obtained. This effect is related to the high affinity of the functionalized nanoparticles with water and the presence of amino and hydroxyl groups that increase the PWF [51,56]. 

Other copper-based nanocomposites have also been studied for the modification of PES-based UF membranes. On the one hand, Szymański et al. reported the influence of the addition of copper-modified titanate nanotubes (Cu/TNT) on the permeability, antifouling and antibacterial properties of PES membranes [49]. A notable increase in the permeability of the membranes modified by the incorporation of Cu-NPs with respect to the pristine membrane was observed. Furthermore, the addition of Cu/TNT in the PES membrane improved its antibacterial properties against bacteria, *S. epidermidis* and *E. coli*. This property was related to the presence of Cu-NPs. However, no significant changes in antifouling property were recorded.

On the other hand, the incorporation of Halloysite nanotubes loaded with copper ions (Cu^2+^-HNTs/PES) in PES membranes was studied by Yifeng Chen et al. [52]. The contact angle decreased with the increasing of the Cu^2+^-HNTs concentration, which indicates that the membrane surface became more hydrophilic after adding Cu^2+^-HNTs. Besides, the experimental results showed that the PWF of hybrid membranes increased with the addition of Cu^2+^-HNTs and their antibacterial performance against gram negative bacteria (*E. coli*) and gram positive bacteria (*S. Aureus*) was significantly higher with 100% of efficiency. Nevertheless, the antifouling properties and stability of these composites in the modified membranes were not evaluated. Alternatively, Gul et al. developed polyethersulfone/cellulose acetate membranes with the incorporation of Ag_2_ONPs (PES-CA-Ag_2_O) by the inversion phase process. Cu-NPs were grown on the surface of the nanocomposite membrane (Cu^0^ @ PES-CA-Ag_2_O) [53]. The addition of metallic NPs exhibited better hydrophilicity performance and PWF performance relative to PES-CA membranes. These capabilities are highly recognized for Cu-NPs incorporation and are attributed to the formation of a hydration sphere on the membrane surface. Moreover, higher antifouling properties on modified membranes were observed with respect to PES-CA membranes with higher BSA rejections values. Thus, the adsorbed protein foulant could be easily removed from the hydrophilic surface of membranes by simple hydraulic cleaning. Accordingly, the incorporation of Cu-NPs showed a similar trend for antibacterial efficacy where the Cu^0^ @ PES-CA-Ag_2_O membrane has a greater antibacterial capacity.

The previous results confirm the hydrophilic and antibacterial effect that copper-based nanocomposites confer on modified PES-based UF membranes. Furthermore, they reveal an incipient strategy to obtain antifouling membranes based on Cu incorporation in PES matrix through: (1) polymer with hydrophilic functional groups along with Cu-NPs that are used as additives within the casting solution in order to promote the orientation of hydrophilic groups and migration of copper to the membrane surface, (2) the functionalization of Cu-NPs surface with functional groups that allow to improve the adaptability of the surface of these nanoparticles (hydrophilic) with a polymer surface (hydrophobic), as well as to increase the stability of dispersion in the media. Both strategies should be required to estimate the optimal additive concentration that has the least impact on viscosity of casting solution in order to not negatively affect the porous density of the membrane, which can be studied by modelling methods. Methodologies similar to those used on PES modification [49,52,53] have been employed by other researchers for the modification of PVDF-based UF membranes. Zhao et al. worked on the incorporation of hydrophilic GO and antibacterial CuO-NPs in PVDF membranes by inversion phases process [54]. CuO/GO modified membranes presented a lower contact angle compared to the pristine PVDF membrane, showing that the surface hydrophilicity was substantially higher. Moreover, the modified membrane presented a higher BSA rejection, better FRR (%) and a significant anti-biofouling activity compared to the PVDF membrane, proving that the modification of hydrophilic nanofiller allows membranes to be more fouling resistant.

Isawi et al. modified PVDF membranes via an ultrathin coating surface layer of a dilute poly (vinyl alcohol) (PVA) aqueous solution in order to provide sufficient hydrophilicity and a reduced surface roughness. Moreover, they incorporated CuO-NPs in the PVDF supports using a phase inversion method [57]. It can be seen from the water flux and the HA rejection that the modified PVDF/PVA/CuO membrane exhibited a significant improvement in performance compared to the PVDF and PVA/PVDF membranes. Nevertheless, the antifouling and anti-biofouling effect of this modification have not yet been reported. In addition, the incorporation of CuO-NPs functionalized with antibacterial polymer in PVDF membrane has been used as alternative strategy to improve the dispersion of CuO-NPs in PVDF polymeric matrix, and the anti-biofouling membrane properties. Thus, Saraswathi et al incorporated CuO-NPs functionalized with polyhexamide (P-CuO NPs) in PVDF membranes by adding them into casting solution during the membrane synthesis by phase inversion method [81]. The PVDF/P–CuO nanocomposite membranes showed high pores content on the surface top with interconnected macro-voids, and this behavior improved with the increase concentration of P-CuO NPs in the membrane matrix. The incorporation of the highest P-CuO concentration (3 wt%) produced the most hydrophilic membrane with increased water permeation, foulant separation and antifouling behavior (see Table 5, entry 13). The PVDF/P-CuO membranes showed high antibacterial response by halo-zone test. However, the anti-biofouling properties should be evaluated under long operation times with real water conditions in order to confirm the applicability potential of this modification in water treatments.

On the other hand, hydrogels with copper ions and hybrid nanogels have been used for modifying PVDF-UF membranes. In this way, Gao et al modified the PVDF membrane surface with ultrathin Cu^2+^/alginate hydrogel multilayer with controllable thickness at the nanometer scale via a LbL self-assembly method [82]. The Cu^2+^-PAA-g-PVDF modified membranes showed a biomimetic superhydrophilicity, underwater superoleophobicity, and antifouling ability for crude oil. It is capable of efficiently separating crude oil in water emulsion with a high-water flux of 1230 L m^−2^ h^−1^ bar^−1^, an ultrahigh efficiency of 99.8%, and an outstanding antifouling and cyclic ability. However, the water flux decreased with the increasing of layer number deposited on membrane surface. While Padmavathy et al used hybrid nanogels (PPE-CuO), composed of PPE (polyphosphoester) and CuO-NPs, with inherent antifouling and antibacterial properties, to modify polyvinylidene fluoride–styrene maleic anhydride (PVDF/SMA) surface membranes by grafting method [83]. The grafting of hybrid nanogels PPE-CuO on PVDF/SMA membrane surface produced membranes more hydrophilic with high antifouling properties achieved by electrostatic repulsion between hydrophilic membrane surface and hydrophobic foulants such as BSA and HA. In addition, the PPE-CuO/PVDF/SMA membranes showed water flux increased by the high porous formation, and high bactericidal effect against *E*. *coli* by isolated tests. Hence, future studies could be focused to test the modified membranes under real water conditions in order to achieve the optimization of different parameters to scaling up this modification in water treatments.

Thus, although studies on modifying PVDF incorporating copper nanoparticles are minor compared to those incorporating PES, it has been possible to observe that the strategies employed to modify PES have been redirected towards modifying PVDF in order to improve the same properties described above for PES. Sundaram et al. [58] modified the UF membrane using PEI and studied its modification by incorporating CuO-NPs coated with an antibacterial polymer and the poly (hexamethylene biguanide) hydrochloride (PHMB-c-CuO) through the inversion phase process. Decrease in contact angle and an increase in the performance of the PWF are measured upon increasing the PHMB-c-CuO concentration in the PEI membrane. In addition, all modified membranes showed better antifouling performance and FRR (%) with respect to the pristine PEI membrane evaluated to BSA, HA, and oil-water. Thus, the membrane (PHMB-c-CuO) is a potential antifouling membrane.

In contrast, Xu et al. deposited PEI onto a microporous PAN membrane surface via electrostatic self-assembly followed by immobilization of copper (II) ions on the membrane surface [59]. It is interesting to note that the PEI deposition caused a decrease in surface hydrophilicity and permeability performance of the modified membranes. This effect may be related to the blocking of the pores from the PAN by the deposition of the PEI layer. Thus, PEI layer had a detrimental effect on the filtering performance of the modified membranes. Nevertheless, the PAN-PEI-Cu membrane exhibited an antibacterial efficiency against *E. coli*, higher than PAN-PEI membrane, showing that copper indeed acted as a strong biocide.

Alternately, to improve the interfacial affinity and anti-fouling properties of UF membranes polyphenylsulfone (PPSU) based, Arumugham et al. incorporated nano sheets of graphitic carbon nitride (g-C_3_N_4_) doping with CuO (CuO/g-C_3_N_4_) through the phase inversion process [43]. The modified membranes showed an increase of the hydrophilic and PWF capacities with respect to the PPSU membrane. These results are associated to the migration of CuO/g-C_3_N_4_ sheets to the membrane surfaces during the inversion phases process, since they promote more hydrophilic active sites (Cu-O), which could form a hydrated pore structure via hydrogen bonding with interfacial water molecules. Furthermore, all membranes exhibited considerably high protein rejection at around 96%. FRR (%) showed a rise with increasing CuO/g-C_3_N_4_ concentration in the PPSU membrane. However, an increase in the concentration of CuO/g-C_3_N_4_ showed a decline in water flux. This could have happened since beyond an optimum concentration of CuO/g-C_3_N_4_ sheets the membrane porosity reduces, thickness marginally increases, and a dense structure of the membranes is formed diminishing the water permeation rate, which should be considered in future research. Finally, the study of grafted of poly(4-vinylpyridine) (P4VP) onto PSF membranes via surface-initiated atom transfer radical polymerization (SI-ATRP) and then immobilized copper (II) ions on the modified membrane was developed by Qiu et al. [60]. This modification exhibited important antibacterial effects of the modified membrane given the incorporation of Cu against *E. coli* with an efficiency of 100%. However, filtering performance, permeability, hydrophilicity and antifouling effects have not yet been reported.

In summary, UF membranes have been modified with the incorporation of Cu-NPs, CuO-NPs, Cu_2_O-NPs or Cu-composites, mainly during the phase inversion process and to a lesser extent, by using different surface modification techniques, such as chemical grafting and surface coating. The main focus has been on improving the hydrophilicity, the permeability performance as well as the antifoulant and antibacterial properties. During the phase inversion process, the hydrophilicity of copper-based NPs and their capacity to migrate to the membrane surface increase the porosity of the matrix and the hydrophilicity of the material. However, changes in the hydrophilicity and permeability of the membrane as well as the stability and good distribution of copper-based NPs within the polymeric matrix, were dependent on the quantity of incorporated NPs. Moreover, the influence of the size and morphology of these NPs in the UF membranes performance looks relevant, though the trend about it is still unclear. Promising strategies to improve the stability and good distribution of copper-based NPs incorporated in UF membranes have been proposed, especially the functionalization of Cu-NPs surface with functional groups that allow the adaptability of the surface of these hydrophilic nanoparticles with the hydrophobic polymer surface. In addition, the use of polymers with hydrophilic functional groups have been proposed, along with Cu-NPs as additives within the casting solution in order to promote the orientation of hydrophilic groups and migration of copper to membrane surface. However, changes of casting solution viscosity given the use of additives should be addressed in order to find the optimal additive concentration without negatively affecting the porous density of membrane. This can be potentially considered by modelling studies.

### 3.5. Microfiltration (MF) Membranes

Microfiltration membrane separation processes are a well-known technology for the removal of micrometric sized contaminants from water such as particles, bacteria, pathogenic agents, proteins, and organic-inorganic matter. Hence, MF are able to remove a wide range of small suspended pollutants in an approximate scale of 0.1–10 μm thanks to their pore diameter that ranges from 0.1–5 μm, making the MF implementation more versatile regarding real industrial applications [19,159]. In this vein, there are numerous polymers matrix that have been reported as being useful MF membrane materials, among which the most common are PES, PSf and PVDF.

The areas of MF application related with water purification are mainly on pretreatments associated with wastewater treatment and desalination. The direct use of MF in desalination is unsuitable by the membrane’s conventional characteristics, where the most useful implementation is when it is used as a pretreatment on the removal of bigger impurities prior to the use of high pressure driven techniques such as RO or NF. Wastewater treatment is undoubtedly the major trend in MF technologies, handling more than 58% of research outputs in the area in the last decade [19]. The MF membranes are especially useful in the initial stages of filtration, so that they have caused a growing scientific interest regarding efficiency, cost-effectiveness and diversity of the membranes with potential applications. 

A specific trend for wastewater treatment, which allows improvements regarding fouling, is the fabrication and chemical modification of MF membranes through a wide variety of techniques [160]. In this regard, MF membrane modification integrates various strategies commonly used in the modification of polymeric materials, combining versatile physicochemical modification techniques with manufacturing methods. Studies have proposed surface grafting, surface coating and polymer blending match with various methods to prepare the polymeric membranes such as stretching, track-etching, sintering, phase inversion, electrospinning and solution coating, creating specific methodological routes to achieve certain properties [19,159,160,161]. Moreover, the introduction of metallic/oxide nanoparticles is a proven technique to enhance the hydrophilicity of this polymer matrix, and in some cases as in copper nanoparticles’, this improvement can include anti-biofouling effect. Thus, these polymer nanocomposite membranes are an interesting approach, even when they become an ambitious trend when they are related to the membrane bioreactors (MBR). This means that they are used with an advanced microfiltration technique that combines biological degradations processes and physical rejection in a single step, led by process intensification studies [20].

Therefore, the use of copper species in MF could lead to an advanced materials development approach in MF. Despite these potential advantages, reports about copper-related modifications in MF are limited and maintain large methodological differences among them, having only in common the search for antifouling properties along with some minor methodological details, as shown in Table 6. Some of them take advantage of the bactericidal capabilities of copper and synthesizing materials that could potentially be used in MBR applications due to their antibacterial activity combined with MF.

In this regard, the studies will be descriptively analyzed based on the strategies, methodologies and results of the copper-containing modifications to improve hydrophilicity, reducing the membrane fouling effect, and allowing antibacterial activity in some cases. Regarding the specific search for antifouling properties, Liu et al. [44] explored the use of copper oxide nanosheets as a nanoparticle load in an alternative co-fluoropolymer matrix poly(vinylidene fluoride-co-hexa-fluoropropylene) (PVDF-HFP) using a combined synthesis method that included electrospinning, heating sintering and hydrothermal processes. The resulting CuO-nanosheet loaded polymeric matrix claimed to reach superhydrophilicity and high mechanical flexibility, due to the nanofibrous stratified structure (polymer fibers/inorganic sheets). The filtration test showed a high effective separation (99.89%) of suspended polymeric particles of polystyrene (PS-30 μm) maintaining 98.1% of water flux after 60 min being operational, suggesting a good antifouling effect against hydrophobic particles. The antifouling effects were attributed to the high hydrophilicity and roughness achieved with the CuO modification. Along with the acceptable flux properties reported (2360 $L/h\;m^{2}$), the study reported good mechanical properties for a polymer nanocomposite, which makes it a good candidate for real MF applications. Despite these good reported results, there is a lack of testing necessary to prove the potential wide-field applications, such as essays of biofouling resistance or fouling tests against other major foulants like proteins or natural organic matter (NOM), usually present in either waste-water or industrial residual waters. Finally, the potential antibacterial or bacteriostatic effect of CuO-nanosheets could be a hidden advantage of this material.

Equally, Park et al. [45] modified a fluoropolymer matrix of PVDF with amine-modified multiwall carbon nanotubes (MWCNTs) mediated by a complexation copper mediated reaction through an atomic transfer radical addition (ATRA) using the hydrophilic Cu[DNDP]_3_MWCNT-EA complex as a non-removable intermediary. They also employed the non-solvent induced phase separation process (NIPS) for the membrane preparation. A water flux increment in the modified membrane was observed with respect to the bare polymer matrix by 155.3% and a high rejection of the artificial foulant BSA protein (98%) with lower absorption than the original PVDF. The FRR remained at 92.7% after the fouling performance. The authors attributed the good performance results and the antifouling activity to the formation of a hydration layer facilitated by the interaction of the highly hydrophilic Cu[DNDP]_3_MWCNT-EA complex present in the modified PVDF. Additionally, the authors verified the attachment of the Cu[DNDP]_3_MWCNT-EA particles, guaranteeing stability for up to 7 days under pressure. These results put the membrane as a good alternative to PVDF, due to its antifouling properties, tested with a standard protein. The increase in hydrophilicity due to the cupric complex and its hydrogen-bridge interactions with the surrounding water is an interesting aspect providing high hydrophilicity. It is possible that the membranes may have additional bacteriostatic behavior which should be verified by an antibiofouling test. Despite the good results in both works, the synthesis process is relatively complex, and the reproduction of combined techniques could be a methodological challenge for real field implementation, especially when the main use is microfiltration pretreatment.

Some works have also looked for the bactericidal effect of copper together with antifouling properties, synthesizing potentially useful membranes for MBR-type processes. For example, Dasari et al. [46] prepared a non-woven electrospun membrane using polylactic acid (PLA) functionalized with copper-loaded sepiolite fibrillar particles negatively charged. The resulting membranes were tested against bacterial biofouling using *S. cerevisiae* and *P. putida* microorganisms, measuring the resultant biomass after operational 24 h/48 h using adenosine-5-triphosphate (ATP) determination. The results show significant depletions in the active biomass in the presence of Cu/Sep particles. For both essays the antibacterial activity was attributed to the Cu^2+^ ions released from the fibrillar hybrid matrix by dissolution processes. Water permeability was enhanced for Cu/Sep membranes with respect to PLA. The permeability of copper containing fouled membranes showed a significant decrease of almost 50% in the water flux, which could be a considerable limitation for the lifespan of the membranes. It should be noted that despite the considerable load of copper on the membranes, in this case, the copper could be trapped within the stratified system of the clay sepiolite particles, limiting direct contact and therefore the generally attributed increase in hydrophilicity. Naturally, the dissolution phenomena allowed the bactericidal action of copper ions, but they did not prevent the attachment of dead bacteria on the membrane, resulting in a formed biofilm with possible implications on the membrane performance. 

Another similar work was reported by Ghalamchi et al. [47], which showed the preparation of MF membranes by means of embedding Ag_3_PO_4_/ZnAlCu nanolayered double hydroxide (NLDH) into a polymer matrix of PES. The methodology used combined co-precipitation techniques for de NLDH synthesis and NIPS to fabricate the non-woven membranes. The main objectives were to increase the hydrophilicity and allow antibacterial activity in the membrane adding a photoactive material. The performance of the membranes was improved in terms of pure water flux from 229 $L/h\;m^{2}$ in PES to 269.5 $L/h\;m^{2}$ max. in the modified membranes. The antifouling properties were tested against the standard BSA protein, showing a high BSA rejection (around 95%) and an FRR index maximum value of 72.4%. Additionally, the antibacterial and antibiofouling activity was assessed probing the membrane for filamentous bacteria activated sludge filtration and both disc diffusion and colony forming techniques (*E. coli S. aureus and B. anthracis* were model microorganisms). Accordingly, the FRR value increased from 64.2% to a maximum of 89.6% in the sludge processing, along with a clear qualitative antibacterial effect in contrast with the bacteria in the halo assays and quantitative decrease in the colony counts. The authors attributed the combined antifouling enhancement to an increase in the hydrophilicity due to the copper based NLDH embedding the structure. The antibacterial effect was attributed mainly to the photo-induced ROS generation in the NDHL interface and the controlled leaching of silver and copper ions. The result confirmed the potential use of these membranes in MBR applications. The main concern within this work is the high accumulation of doping agents on the surface, and the consequent loss of homogeneity of the material, which could lead to a low reproducibility of manufacturing by NIPS method.

Finally, Fazullin et al. [48] prepared antibacterial membranes by directly treating Nylon commercial membranes (PA, Phenex 0.45 μm) with different solutions with active antibacterial agents. Among the latter, they used copper sulfate through simple dead-end filtration setup, with dynamic recirculation of solution and aging of functionalized membranes. The copper content of the commercial membranes after treatment reached 3.8% wt, with a non-uniform distribution of relatively big particles (0.1 to 5 μm) negatively charged on the surface. The membranes showed bacteriostatic properties and inhibition of organism growth (510 CFU mL^−1^ vs. 6 CFU mL^−1^). The performance of the membranes treated with CuSO_4_, showed a mild decrease (9.3 to 8.8 cm^3^/cm^2^ min) in the pure water flux determination. From this study it is important to highlight both the simplicity of the functionalization technique and the good one-off results. Consequently, future work should consider the evaluation of the potential high mass release of copper into pure water or the potential antifouling effect against a minimum running time in microfiltration.

In summary, reports about copper-related modification in MF are limited and, in most cases, the searched common benefit is related to obtaining antifouling properties. Various strategies used in the modification of these polymeric materials imply the combination of different methods such as Electrospinning-Heating-Hydrothermal, Blending-Electrospinning, ATRA-NIPS, Co-precipitation-NIPS, and Immersion-Aging. Overall, these modifications have improved the hydrophilicity, reduced the membrane fouling effect, and have allowed antibacterial activity. Despite these good specific results, there is a lack of testing necessary to prove the potential wide-field applications. The synthesis processes/modification can be relatively complex, and the reproduction of combined techniques could be a methodological challenge for real field implementation. For instance, concerns such as high accumulation of doping agents on the surface and loss of homogeneity of the material are important. Moreover, the impact of copper release into water flux and the potential antifouling effect under real microfiltration conditions are new focus to be considered. Finally, it is pivotal to note that these materials could be potentially used in MBR applications, due to their antibacterial activity combined with MF. Thus, an ambitious opportunity related to membrane bioreactors (MBR) could be considered, since it is an advanced microfiltration technique that combines biological degradations processes and physical rejection in a single step. 

## 4. New Challenges and Perspectives 

### 4.1. Improvements in Membrane Modification Procedures for the Effective Incorporation of Copper Species

As discussed above, novel copper-modified membranes are viewed as materials that can potentially improve the performance of membranes with respect to selectivity, water flux and anti-fouling/biofouling properties, among other features. However, significant advancements of material modification procedures are still needed, mainly in order to advance towards the industrial scaling up of these modified membranes that keep the modification effectiveness over time. 

Relevant drawbacks can be drawn from the incorporation of copper into membrane matrices, which currently remain unsolved. For instance, the formation of defects, pore blocks and nanoparticles agglomeration phenomenon on modified membranes during their fabrication. The low modification stability, the uncontrolled copper ion release or leaching of the incorporated copper material are examples of equally important and unsolved issues in this matter. In this regard, new challenges related to the modification approach directed to solve the already described difficulties have been identified. Two major strategies to incorporate copper in/on polymeric membranes for water treatment have been explored. The first one, by the incorporation of copper-based materials during the synthesis process of the membrane, and the second one, by the incorporation of superficial modification of the membranes using different techniques such as coating surface, grafting, and layer by layer approach. The second strategy has gained significant attention, yet their inherent issues may trigger limitations to produce effective copper-modified membranes. Thus, from our point of view, some of these concerns can be addressed with strategies in each technique to achieve effective copper incorporation into the membrane. 

For instance, the coating surface approach has been widely used to incorporate copper based materials on RO, NF membranes and UF membranes, producing in most cases modified membranes with low modification stability and loss of effectiveness by early depletion of built-in copper, limiting its application during long operating times. In order to solve these concerns, studies suggest the use of inexpensive copper complexing agents to stabilize the copper on the membrane and control the capacity of copper ion dissolution. For this purpose, some alternatives are the stabilizing agents with heteroatoms that have high affinity with copper, such as nitrogen and/or sulfur containing functional groups. On the other hand, the grafting approach that has been applied for modifying RO and MF membranes achieves copper-modified membranes with long-lasting performance, mainly regarding the antibacterial effect. In general, this is due to the slow release of copper ions produced by the chelation effect of different linker agents on copper ions, but in some cases, the membrane flux could be affected. Thus, the use of copper chelating agents with hydrophilic features is likely to improve the copper retention and the hydrophilicity of the membrane. 

On the contrary, the layer-by-layer approach has been used to a lesser extent to obtain RO, FO and NF modified membranes. In this regard, systematic studies to find out the optimal conditions that allow to achieve a high load of copper by a lower layer numbers deposition are required in order to avoid the water flow falling. New perspectives and future work on the production strategies of copper-modified membranes can also be analyzed for each membrane type. Therefore, our point of view to address each case is presented as follows. For RO membranes, biofouling mitigation is the main reported target to modify TFC-RO membranes when incorporating copper. According to the analysis, although different experimental techniques (coating, grafting, layer by layer assembly and interfacial polymerization processes) have been used to promote significant anti-biofouling effect, these have a number of disadvantages associated with the techniques themselves, related to the stability of modification, loss of effectiveness over time, and limitations in long-lasting operating times. Then, it is possible to conclude that there is not still a clear way to modify TFC-RO membranes with copper. The immobilization of copper materials during interfacial polymerization processes and the grafting approach are envisaged as ways that have a wide research field to develop. On the one hand, the immobilization of copper materials within a polymer matrix through their incorporation during IPP is a strategy that has gained attention in recent years. However, the relation between the incorporated copper-material and IPP conditions, such as monomers concentration, solvents, interaction times, and copper nanomaterials dispersion strategies, are important factors that need to be addressed in order to avoid surface defects on the active layers and/or the copper particles agglomeration, phenomena that could severely affect the surface properties and performance of the membrane material. Hence, this approach has a great potential to produce copper modified TFC-RO membranes with tunable properties, but it also might require a long way to achieve its industrial application. In the second case, the grafting approach represents a good alternative. As it was mentioned, the use of linker agents with copper chelation affinity should stabilize the release of copper ions from the modified membrane, but a possible detrimental effect on membrane flux could be observed. Thus, as a guideline, new grafting strategies might include the use of branched polymers and graphene oxide with amine or thiol functional groups in order to achieve an efficient chelation of copper and an increase in the hydrophilicity of the membrane.

As for FO membranes, although there has been increasing attention to their production over the last decade, it has been mainly directed at overcoming major problems such as fouling/biofouling susceptibility and low chemical resistance. There are only few reports of copper-modified membrane production to solve these serious bottlenecks. Surprisingly, the available research is totally dedicated to improving structural parameters to avoid internal concentration polarization adverse effects, relegating to a second tier the most valuable property of copper derivatives: antibacterial activity. According to the reports, the use of MOFs is undoubtedly a valid option in terms of enhancement of compatibility between organic-inorganic matrices and active reservoirs of metallic species (e.g., Cu), in addition to their properties as a porosity/defect forming agent to increase the total water flow. However, agglomeration, high solubility, and uncontrolled release of ions into the environment are matters to be solved. Alternatives that should include both the synthesis of stable Cu-MOF in aqueous solution and high pH range, and the interfacial functionalization of the same with a stabilizing agent to achieve its homogeneous incorporation in the membrane can be proposed. Alternatively, the use of Cu/CuO-type nanoparticles stabilized by surface functionalization with membrane-like organic agents could be explored as a lower-cost option that could satisfy the requirements of porosity, hydrophilicity and antibacterial activity in a single synthesis step, but with significant technical challenges to be solved. Finally, the bio-inspired techniques such as the Mussel methods, including Cu species, could have a major impact on the synthesis of new membranes, but a major synthesis parameter adjustment is definitely needed to equalize the mechanochemical stability of actual TFC-FO conventional membranes. This milestone is only reachable exploring more case studies and performance modeling approaches.

Regarding NF membranes, the use of copper-based materials in nanofiltration is not far away from the core trend, focusing on improvements of hydrophilicity, antibacterial effect, and porosity characteristics, with two main applications of copper-modified NF membranes well reported: WWT and desalination. Despite the good results on selectivity regarding WWT, mainly against divalent ions and small/medium organic molecules, the modifications show specific weaknesses related with the stability itself with high rate of inhomogeneous pore size. Thus, the recent advances in nanotechnology could be a great complement for future copper-related modifications. Taking into account the new generation of sustainable materials, such as nanocelluloses, and effective methods for functionalization, such as photo-grafting, plasma etching and layer by layer, with functionalization tailoring approaches in both components (membranes and nanoparticles), it is possible to address problems of material inhomogeneity and pore-related low divalent ion rejection. A successful strategy could ensure the material reproducibility and feasibility in front of a potential modular manufacturing in the future. Research efforts in NF for desalination should be redirected to complementary applications due to the inevitable low rejection rates of monovalent ions. This does not mean to stop studies in this area, but designing the materials for pre-treatment stages, combining the advantages offered by the copper species at bactericidal and hydrophilic level, with the selectivity of the NF against divalent salts and polymeric/oligomeric molecules. This could be done through advanced synthesis techniques, which must necessarily include interfacial stabilization developments between the organic phase of the membrane and the inorganic copper species.

Improvements on hydrophilicity have particularly been the main target to incorporate copper materials in UF membranes, yet the anti-biofouling properties have been only superficially studied. Much work related to embedding copper nanomaterials in the casting solution to develop copper modified UF membranes have been reported. However, systematic studies that describe the influence of nanomaterial properties such as morphology, size, and type, on the modified membrane properties have not been explored yet. In addition, the copper nanoparticle functionalization with polar groups such as amine or sulfonic derivatives is envisaged as a potential strategy to improve the interaction between nanomaterial and polymeric matrices. Nevertheless, these studies should be strongly supported by modelling methods aimed at optimizing the concentration of additives, thus avoiding negative repercussions on parameters such as the viscosity of the casting solution and, consequently, on the porous density of the resulting membrane. Finally, biofouling studies on UF membranes that have been modified with copper should be addressed with the aim of taking advantage of antimicrobial copper properties and increasing the added value of the modified membrane.

With respect to the use of copper in MF membranes, developments to date have been limited. In addition to this, these works present a remarkably high degree of complexity in the experimental synthesis, with few coincidences among the methodologies themselves. This may complicate their replicability, viability and cost-benefit ratio compared to the already established commercial membranes for simple microfiltration processes. However, processes such as membrane bioreactor have gained much relevance at the industrial level and require membrane materials with superior properties that combine antibacterial, antifouling, biochemical resistance and durability, which are the main trends in microfiltration advances. Therefore, copper-based membrane modifications could create a research and application niche since they provide several of the characteristics required in MBR. Consequently, it is necessary to adjust the stability problems of the materials so far proposed in MBR to facilitate their correct functionalization with copper species, such as materials based on fibers modified with nanoparticles, compact filaments enriched in active metallic species or non-woven polymeric membranes charged with nanomaterials. This adjustment is necessary to ensure that the lifespan of the material corresponds with its antibacterial activity and its chemical-mechanical properties. A valuable contribution could be the exploration of colloidal copper species mixed with polymeric solutions by electrospinning, along with surface activation treatments of polymeric fibers, for the subsequent loading with inorganic copper species by hybrid interfacial stabilization. This is a clear example of low explored synthesis possibilities with a minimal complexity approach.

### 4.2. Scale Up Implementation and Environmental Impact

The likelihood of industrial scaling up of these membranes modified with copper could be achieved if the loss of modification effectiveness over time is avoided. This is related to several key manufacturing challenges on modification stability improvements, ion release control and leaching of the incorporated copper material, among others observed obstacles, whose membrane modification strategies were described in the last section. However, the development of new copper-modified polymeric membranes will also face the challenge of demonstrating the capabilities needed to replace the existing water treatment membranes. Thus, other key aspects must be considered: (i) long-term performance under real conditions, (ii) feasibility of large-scale production, and (iii) environmental impact assessment. 

The long-term performance of these modified membranes should be determined by keeping the structural characteristics and properties under real process conditions, validating a stable modification and a controlled copper release from the membrane over time. In this regard, the hydrodynamic conditions defined by the membrane module where the copper-based membranes will be installed could determine the copper species leaching rate from the material and their impact on the chemical characteristics of the affluent treated. Industrial systems operate in a steady state with specific flow velocities, so it will be important to be able to predict how long the copper will retain the searched properties in the long term. Mathematical modeling and experimental works to predict the copper leaching and release rate of a steady state membrane module for a specific purpose mimicking real hydrodynamic conditions are required. Currently, there have been limited efforts in this regard. The estimation of this parameter with respect to the time is thus relevant to predict the copper profile expected in the system and the lifespan of the modification. 

On the one hand, structural changes on the modified membrane given the influence of the copper released in a long operation time have been briefly studied. This fact could affect the membrane performance in the long term, and could limit the lifespan of the membrane, even before all the contained copper is released. Thus, the assessment of the morphological and structural modifications on the membrane characteristics by the copper dissolution must be included in further studies. In both fields, the advance on mathematical modeling and experimental studies of copper released from the membrane under real hydrodynamic conditions, including the impact on the membrane characteristics, will be relevant to ensure a successful implementation at a larger scale. 

On the other hand, the prototype modules must still be scaled in size and functionality and validated at the industrial level. For this purpose, production cost at large scale must be considered. The production of copper-modified membranes at large scale involves the ensuring of copper-based material supply. Production costs of these copper materials and membrane manufacturing could be critical points in an economic evaluation. The demand for copper-based materials, mainly copper nanoparticles, is growing, and issues related to the required production or risks to price increase could occur. Furthermore, the massive production of the copper materials to be used in the modification must ensure product quality. For instance, for copper nanoparticle size, distribution and stability are pivotal aspects to be guaranteed in mass production.

Finally, the assessment of environmental impact is closely related to the possibility to estimate the copper concentration profile with respect to time in permeate and retentate flows of a membrane process under real conditions. The concentration results of these estimations must be compared with local regulations. For instance, while 2.0 mg/L is below the World Health Organization (WHO) guidance level for drinking water, the United States Environmental Protection Agency (US EPA) drinking water maximum contaminant level goal for copper is 1.3 mg/L. In addition, desalination plant discharges should have a daily maximum copper limit. For instance, according to the California Ocean Plan, 12 μg/L is required, and an instantaneous maximum of 30 μg/L to meet its objective for protection of aquatic organisms [135]. Thus, these values of maximum concentration of copper in water must be considered for a final implementation. The copper release concentration from modified membranes with Cu-NPs reported previously [85,162] have shown a results ranging from 0.1 mg/L to 2.0 mg/L, depending on NP type, membrane type and modification route used. However, these results have been obtained under batch conditions, where the copper concentration is increased by accumulation in the flask, although the slow turbulence conditions used could have limited the leaching velocity. Therefore, the determination of these values simulating real operation conditions will be relevant to estimate the environmental impact of permeate and retentate generated. In addition, the assessment of the final use or destination of membranes replaced must be conducted to determine the potential environmental impact of the remained copper present in the membranes.

## 5. Conclusions

There is evidence of a significant interest in the use of copper materials to improve different properties on polymeric membranes for water treatment. Different copper materials have been identified, such as metallic and oxide nanoparticles, salts, composites, metal-polymer complexes and coordination polymers, with tunable characteristics that favor their incorporation in polymeric membranes. Thus, modification of microfiltration (MF), ultrafiltration (UF), nanofiltration (NF), forward osmosis (FO) and reverse osmosis (RO) membranes incorporating these copper-based materials have been used to improve different properties for each case. For instance, antibacterial and anti-fouling effects, hydrophilicity increase, improvements of the water flux, the rejection capacity of compounds and structural membrane parameters, and the reduction of concentration polarization phenomena are some remarkably improved properties. Different membrane modification approaches to incorporate copper were recognized. For instance, the incorporation of copper-based materials during the synthesis process of the membrane and the membrane surface modification using physical and chemical surface modification techniques. Moreover, relevant drawbacks given the incorporation of copper into membrane matrices remain unsolved. For instance, the formation of defects, pore blocks and nanoparticles agglomeration phenomenon on modified membrane during its fabrication, including the low modification stability, the uncontrolled copper ion releasing or leaching of incorporated copper material remain inconclusive. 

Thus, strategies are required for each membrane case to achieve an effective copper incorporation on these polymeric membranes through membrane modification procedure improvements. In this regard, novel strategies such as (a) the selection of new additives to form copper-based complex materials, (b) the functionalization tailoring approaches, and (c) the use of copper complexing and chelating agents, might stabilize the copper on the membrane, control the copper ion dissolution, and improve the hydrophilicity of membrane surface, among other benefits. Finally, future industrial and scale up implementation of these modified membranes must consider studies on the long-term performance under real conditions, feasibility of production at large scale. The assessment of the environmental impact through the use of copper must be carefully evaluated in further studies, particularly aspects related to the prediction of copper concentration under real operational conditions and the impact of remained copper content in the membranes after its end-life in the final destination. 

## 6. Patents

Patent (granted). CL201601310, Registry number 58616.

## Figures and Tables

**Table 1:** MBC of copper and copper oxide nanoparticles per bacteria. Extracted from [112].

Strain	MBC (mg/L)	
	Cu-NPs	CuO-NPs
*Staphylococcus aureus* (Golden)	1000	2500
*S. aureus* (Oxford)	250	100
*Escherichia coli* NCTC 9001	250	250
*Proteus* spp.	2500	5000
*Pseudomonas aeruginosa* PAOI	2500	5000

**Table 2:** Summary of TFC-RO membranes modified with different copper material.

Base Polymer/ Membrane	Cu-Type	Modification Method	Performance Characteristics			Ref.
			Antibacterial Efficiency (%)	Anti-Adhesion Efficiency(%)	Flux/Conditions	
Commercial TFC-RO	Cu(OH)_2_	Coating	98 (*E. coli*)	--	Modified = 5.1 L m^−2^ h^−1^ atm^−1^ /NaCl 500 ppm, 7.5 bar	[76]
Commercial TFC-RO	Cu-NPs	Coating	-	87 (*E. Coli*) 96 (*P. aeruginosa*) 79.5 (*S. aureus*)	Modified = 0.34 L m^−2^ h^−1^ /(NaCl 2922.5 ppm, 27.6 bar)	[32]
Commercial TFC-RO	Cu-NPs	Coating	-	89.6	Modified = 2.97 L m^−2^ h^−1^ bar^−1^ /(NaCl 2922.5 ppm, 27.6 bar)	[40]
Commercial TFC-RO	PANI-CuNPs	Grafting	-	-	Modified = 17.2 L m^−2^ h^−1^ /(NaCl 2000 ppm, 3 bar)	[77]
PA/PS	Chitosan-CuNPs	Grafting	99 (*E. coli*)	-	-	[78]
Commercial TFC-RO	Cysteamine-CuNPs	Grafting	85 (*E. coli*)	97	-	[79]
PA/PS	CuO-NPs	Immobilization in PA layer	55 (*E. coli*)	88	Modified = 2.18 L m^−2^ h^−1^ bar^−1^ /(NaCl 1000 ppm, 20.7 bar)	[14]
PA/PS	Cu-NPs	Immobilization in PA layer	>99 (*E. coli*)	97	Modified = 0.42 L m^−2^ h^−1^ bar^−1^ /(NaCl 1000 ppm, 20.7 bar)	[36]
PA/PS	Cu-mPD	Immobilization in PA layer	99 (*E. coli*)	99	Modified = 1.6 L m^−2^ h^−1^ bar^−1^ /(NaCl 1000 ppm, 20.7 bar)	[27]
PA/PS	Cu-NPsCuO-NPsCu-mPD	Immobilization in PA layer	99 (*E. coli*) 99 (*E. coli*) 99 (*E. coli*)	-	-	[85]
PA/PES	CuBTTri-MOF	Immobilization in PA layer	96.6 (*P. aeruginosa*)	-	Modified = 3.38 L m^−2^ h^−1^ bar^−1^ /(NaCl 1000 ppm, 20.7 bar)	[80]
PA/PS	CuO-NPs	PVD	99 (*E. coli*)	-	-	[86]
Commercial TFC-RO	PEI-CuNPs	SSLbL	99 (*E. coli*)	-	-	[87]

**Table 3:** Summary of FO membranes modified with different copper-containing compounds.

Base Polymer/ Membrane	Cu-Type	Modification Method	Performance Characteristics					Ref.
			Antibacterial Efficiency (%)	Contact Angle (°)	Water Permeability	Salt Rejection (%)	S (μm)	
Polyacrylonitrile (PAN)	Cu-BTC MOF	LbL-Phase Inversion	-	29 ± 2	132 ± 10 (L m^−2^ h^−1^)	75 ± 5	190 ± 20	[72]
Cellulose Acetate/Triacetate (CA/CTA)	Cu-BTC MOF	Phase Inversion-Immersion Precipitation	-	55 ± 1	1.41 (L m^−2^ h^−1^ bar^−1^)	-	136	[73]
Polyamide TFC-FO	Cu-BTC MOF 2D	Interfacial Polymerization	-	55 ± 5	3.13 ± 0.30 (L m^−2^ h^−1^ bar^−1^)	50 ± 5	366 ± 41	[74]
Polyamide TFC-FO	Cu^2+^-DOPA complex	One-pot chelating-Mussel	97.6 (*S. aureus*)	50 ± 5	0.7 ± 0.1 (Normalized water flux TFC-FO)	30 ± 10	-	[75]

**Table 4:** Summary of NF membranes modified with copper-containing compounds.

Base Polymer/ Membrane	Cu-Type	Modification Method	Performance Characteristics					Ref.
			Antibacterial Efficiency (%)	Contact Angle (°)	Pure Water Flux	Rejection	Application	
CA/GMA/IDA	Cu^2+^ ions	Homopolymerization-Phase inversion-IMA	27% biofilm area (*P. fluorescens)*	43°	11 $L/h\;m^{2}$ (69 bar)	BSA, Lipase (non-determined)	WWT	[63]
PAN-UF	DOPA-Cu^2+^ /PEI-CuNPs	Mussel-inspired PDA-Two-step deposition and Co-deposition	93.7% (*E. coli*)	35° (max.) 18.5° (min.)	18.2 $L/h\;m^{2}$ bar^−1^ (max.)	18% Na_2_SO_4_, 2% NaCl, ~99% Dyes (0.6–2 kDa)	WWT	[64]
HPAN-UF	PDA-rGO-Cu	In situ reduction-Mussel-inspired PDA	97.9% (*E. coli*)	41.7°	22.8 $L/h\;m^{2}$ bar^−1^	7.4% Na_2_SO_4_, 2.5% NaCl, 99.4% Dye (RB2)	WWT	[42]
HPAN-UF	TA-Cu^2+^	Co-deposition	-	54.5°	52 $L/h\;m^{2}$ bar^−1^ (max.)	22.5% Na_2_SO_4_, 10% NaCl, ~99% Dyes (0.6kDa)	WWT	[65]
PSf/mPIAM	Elemental Cu	Phase Inversion-PVD	*-*	65°	36 $L/h\;m^{2}$ (8 bar)	96% (3500 ppm NaCl)	Desalination	[66]
PSf-poly(PIP)	CuBTC (0.25–0.75 wt%)	Blending-Interfacial Polymerization-Phase inversion	-	59.02° (min.)	5.17 $L/h\;m^{2}$ bar^−1^ (max.)	97.3% MgSO_4_, 36.2% NaCl 99.9% BSA	Desalination	[67]
PSf-poly(PIP)	Cu-Al LDH (0.1 wt%)	Interfacial polymerization	-	37.25° (min.)	7.01 $L/h\;m^{2}$ bar^−1^ (max.)	96.8% Na_2_SO_4_ 95.4% MgSO_4_, 95.6% MgCl_2_, 60.8% NaCl	Desalination	[68]
PES-PVP	CoFe_2_O_4_/CuO-NPs (0.05–1 wt%)	Blending-Phase inversion	-	35° (min.)	34.5 $L/h\;m^{2}$ (max.)	95% Na_2_SO_4_, 72% NaCl, >85% Cu^2+^, Ni^2+^, Pb^2+^	Desalination	[69]
PEI (polyetherimide)	Cu-TNT	Blending-Phase inversion	-	60.3° (min.)	1.25 $L/h\;m^{2}$ bar^−1^ (max.)	80% K_2_SO_4_, 75% NaCl, 45% CaCl_2_	Desalination	[70]
HPAN-CS-SPP	CuSO_4_	LbL-Ionic crosslinking-Deposition	100% (*E. coli*)	53.1° (max.) 25.2° (min.)	74.8 $L/h\;m^{2}$	93.3% Na_2_SO_4_, 77.7% MgSO_4_, 20,3% NaCl, 90% PEG (0.4 kDa)	Desalination	[71]

**Table 5:** A comparative summary of the UF membranes modified with Cu.

**Base Polymer/** **Membrane**	**Cu-Type**	Modification Method	Performance Characteristics					Ref.
			Antibacterial Efficiency (%)	Contact Angle (°)	PWF * (L/m^2^ h)	Rejection (%)	FRR ** (%)	
PES	Cu-NPs	Phase inversion- Immersion precipitation	-	55.3	34.5	86.3 (BSA)	23.8	[28]
PES/SPAES	Cu-NPs	Phase inversion- Immersion precipitation	78.9 (*E. coli*)	52.0	193	98 (BSA)	79	[55]
PES	CuO NPs	non-solvent induced phase separation	-	64.0	869.9 (Kg/m^2^ h)	>97% (BSA)	64.2	[61]
PES	Cu_2_O NPs	Addition in phase inversion	-	72.40	59.5	90 (BSA) 95 (HA) 86 (O/W)	64 73 58	[50]
PES	CuO NPs	Dispersion in phase inversion	-	~62.5	886	-- (BSA)	38.0	[62]
PES	CuO/ZnO Nanocomposite	non-solvent induced phase separation	-	65.5	679	95 (BSA)	50.1	[51,56]
PES	Cu/TNTs	Dispersion in phase inversion	-	-	215 (L/m^2^ h bar)	99 (BSA)	-	[49]
PES	Cu^2+^ ions	Dispersion in phase inversion	100 (*E. coli* and *S. aureus*)	69.8	120.1	-	-	[52]
PES-CA PES-CA-Ag_2_O	Cu NPs Cu NPs	Casting method	10 (*E. coli*) 82 (*E. coli*)	68.5 60.3	72.5 100.2	88.1 (BSA) 89.5 (BSA)	- -	[53]
PSF	Cu NPS	Dispersion in phase inversion	-	69.8	39.5	90 (PEO 200 KDa)	-	[84]
PVDF	Cu_x_O	Dispersion in phase inversion	>90 (*E. coli*)	65.8	23.5	80.7 (BSA)	92.09	[54]
PVDF/PVA	CuO	Dispersion in phase inversion	-	66.4	585	88.3 (HA)	-	[57]
PVDF	P–CuO NPs	Dispersion in phase inversion	-	52.5	152.5	99.5 (BSA) 98.4 (HA)	99.5 (BSA) 98.5 (HA)	[81]
PAA-g-PVDF	Cu^2+^ions	non-solvent induced phase separation and Layer by layer self-assembly	99.1 (*E. coli*)	~0	-	99.8 (oil-in-water emulsions)	83.3 ***	[82]
PVDF/SMA	CuO-PPE NPs	Grafting	98 (*E. coli*)	54.5	1300	~85 (BSA)	-	[83]
PEI	PHMB-c-CuO	Coating surface	-	60.7	192.5	98.2 (BSA) 97.4 (HA) 98.8 (O/W)	99.5 98.5 98.6	[58]
PAN/PEI	Cu^2+^ ions	Coating Surface	71.5 (*E. coli*)	47.7	594 (L/m^2^ MPa)	99 (HA)	-	[59]
PPSU	CuO/g-C_3_N_4_	Phase inversion	-	53	202	96 (BSA)	79	[43]
CMPSF/P4VP	Cu^2+^ ions	Grafting	100 (*E. coli*)	--	-	-	-	[60]

* PWF: Pure Water Flux; ** FRR: Flux Recovery Ratio; *** In the second separation processes, after washing of modified membrane.

**Table 6:** Summary of MF membranes modified with copper-containing compounds.

Base Polymer/ Membrane	Cu-Type	Modification Method	Performance Characteristics			Ref.
			Antibacterial Efficiency (%)	MF Performance H_2_O Pure	FRR Max.	
PVDF-HFP	CuO-Nanosheets	Electrospinning-Heating-Hydrothermal	-	2360.19 $L/h\;m^{2}$	98.1%	[44]
PVDF	Cu[DNDP]_3_MWCNT	ATRA-NIPS	-	2137 $L/h\;m^{2}$	92.7%	[45]
PLA	Cu(26 wt%)/Sepiolite	Blending-Electrospinning	85 (*S. cereviciae*) 35 (*P. putida*)	16.4 $L/h\;m^{2}$	50.0%	[46]
PES	Ag_3_PO_4_/ZnAlCu-NLDH	Co-precipitation-NIPS	*E. coli* *S. aureus* *B. anthraus*	269.5 $L/h\;m^{2}$	89.6%	[47]
Nylon (PA)	CuSO_4_/Cu-NPs	Immersion-Aging	~100 (*Gram +/− mix.*)	8.80 cm^3^ cm^−2^ min^−1^	-	[48]

## Supplementary Materials

The following are available online at https://www.mdpi.com/2077-0375/11/2/93/s1, Prisma checklist, Prisma flow diagram and SRProtocol.

## Abbreviations

MF	Microfiltration
UF	Ultrafiltration
NF	Nanofiltration
FO	Forward Osmosis
RO	Reverse Osmosis
Ag	Silver
Cu	Copper
Fe	Iron
GO	Graphene Oxide
TiO_2_	Titanium Dioxide
Zn	Zinc
NPs	Nanoparticles
Cu-NPs	Copper Metallic Nanoparticles
CuO-NPs	Copper Oxides Nanoparticles
Cu-Al LDH	Copper-aluminum Layered Double Hydroxide
CuBTC	Copper Benzene-1,3,5-tricarboxylate
CuCl_2_	Copper Chloride
Cu^2+^-HNTs	Halloysite nanotubes loaded with copper ions
Cu-MPD	Cu-meta-phenylendiamine Oligomers
Cu(NO_3_)_2_	Copper Nitrate
Cu(OH)_2_	Copper Hydroxide
CuSO_4_	Copper Sulfate
Cu/TNT	Copper-modified Titanate Nanotubes
rGOC	Reduced Graphene Oxide-copper
TA-Cu^2+^	Tannic Acid-cupric Acetate Complex
ATP	Adenosine-5-triphosphate
BSA	Bovine Serum Albumin
MBC	Minimum Bactericidal Concentration
MBR	Membrane Bioreactors
MIC	Minimum Inhibitory Concentration
NOM	Natural Organic Matter
PBS	Phosphate-Buffered Saline
ROS	Reactive Oxygen Species
CA	Cellulose Acetate
Catechol	1,2-dihydroxyphenyl
CCTS	Carboxylate Chitosan
CS	Chitosan
CTA	Cellulose Triacetate
CTAB	Cetyltrimethylammonium bromide
Cys	Cysteamine
GA	Glutaraldehyde
SI-ATRP	Surface-initiated Atom Transfer Radical Polymerization
SSLbL	Spin-assisted Layer-by-layer Self-assembly
DS	Draw Solutions
FS	Feed Solution
FRR	Flux Recovery Ratio
ICP	Internal Concentration Polarization
IMA	Immobilized Metal Affinity
MWCO	Minimal Molecular Weight Cut-off
Gallol	1,2,3-trihydroxyphenyl
g-C_3_N_4_	Graphitic Carbon Nitride
GMA	Glycidyl Methacrylate
HA	Humic Acid
HEPES	4-(2-hydroxyethyl)-1-piperazine ethanesulfonate
IDA	Iminodiacetic Acid
LDH	Layered Double Hydroxide
MPD	m-phenylendiamine
MOFs	Metal Organic Frameworks
MWCNTs	Multiwall Carbon Nanotubes
NLDH	Nanolayered Double Hydroxide
P4VP	poly(4-vinylpyridine)
PA	Polyamide
PAA	Poly(acrylic) Acid
PAN	Polyacrylonitrile
PANI	Polyaniline
PDA	Polydopamine
PDVF	Poly (vinylidene fluoride)
PEI	Polycation Polyethylenimine
PES	Polyethersulfone
PHMB	Poly (hexamethylene biguanide) Hydrochloride
PLA	Polylactic Acid
Ply-PIP	poly (piperazineamide)
PPE	Polyphosphoester
PPSU	Polyphenylsulfone
PS	Polystyrene
PSf	Polysulfone
PVA	poly(vinyl alcohol)
PVD	Physical Vapor Deposition
PVDF-HFP	Poly (vinylidene fluoride-co-hexa-fluoropropylene)
SA	Sodium Alginate
SPAES	Sulfonated Poly (aryl ether sulfone)
SPP	Polyphosphate
TFC	Thin-Film Composite
TMC	Trimesoyl Chloride
TNT	Nanotubes
ALDS	Atomic layer deposition
ATRA	Atomic Transfer Radical Addition
IPP	Interfacial Polymerization Process
LbL	LbL, Layer by Layer
NIPS	Non-solvent Induced Phase Separation
O/W	Oil/Water
PWF	Pure Water Flux
S	Thickness
US EPA	United States Environmental Protection Agency
WHO	World Health Organization
WWT	Wastewater Treatment
X	Tortuosity/porosity
