# Supplementary material for: Copper-Modified Polymeric Membranes for Water Treatment: A Comprehensive Review
Source: Membranes (Basel). 2021 Jan 28;11(2):93. doi: 10.3390/membranes11020093 (PMC7911616; doi:10.3390/membranes11020093)
Supplement: Supplementary file 1 [file membranes-11-00093-s001.zip › membranes-1085993-supplementary/Supplementary Information/SRProtocol-SV_AG.docx]

Review Protocol: Copper-modiﬁed polymeric membranes for water treatment: a comprehensive review

# Team Information

| **Project Lead** | Andreina García |
| --- | --- |
| **Research Team Members** | Andreina García^1,2*^ , Bárbara Rodríguez^2*^ ,Hugo Giraldo^2^, Yurieth Quintero^2^, Rodrigo Quezada^2^, Natalia Hassan^3^ and Humberto Estay^2^ |
| **Date** | January, 2020 |
| **Institution(s)** | ^1^ Mining Engineering Department, FCFM, Universidad de Chile, Santiago 8370451, Chile.  ^2^ Advanced Mining Technology Center (AMTC), Universidad de Chile, Santiago 8370451, Chile.  ^3^ Programa Institucional de Fomento a la I+D+i, Universidad Tecnológica Metropolitana. Ignacio Valdivieso 2409, San Joaquín, Santiago 8940577, Chile. |

# Background

*Describe the population and condition or phenomenon of interest and contextualize it. In other words, describe what this review is about.*

| This manuscript is an extensive review of the progress of copper-modified polymeric membranes for water treatment application. The most notable use of copper materials, (metallic and oxide nanoparticles, salts, composites, metal-polymer complex, coordination polymers) for modifying microfiltration (MF), ultrafiltration (UF), nanofiltration (NF), forward osmosis (FO) and reverse osmosis (RO) membranes is examined. Advantages and limitations of the different membrane modification approaches for the effective copper incorporation are critically analyzed in each case.  Moreover, the benefits of the copper incorporation into these matrices on the membrane properties, such as, selectivity, water flux, anti-fouling/biofouling effect, among others, are remarked, and the relevant detriments as hinders to be solved, are described. For instance, defect formation, pore block and nanoparticles agglomeration phenomenon on modified membrane during its fabrication, moreover, the low modification stability, the uncontrolled copper ions release or leaching of copper material out of the composite, were identified. Thus, strategies and material modification procedure improvements to achieve an effective copper incorporation on these polymeric membranes and solve these hinders, are offered. Furthermore, perspectives about scaling-up implementation considering aspects such as long-term performance under real conditions, feasibility of mass production, and assessment of the environmental impact of the use of copper are presented. |
| --- |

# Objective

*Describe the justification for this review. In other words, describe why this review/the information it collects is important.*

| In the last years detailed reviews of the current developments in the use of polymeric nanocomposite membranes for purifying water, RO, NF, UF, MF and MD have been reported. In these cases, analysis of a significant number of nanomaterials and the different methods used for manufacturing these nanocomposite membranes, have been presented, where the anti-biofouling effect is the core focus to attend. Moreover, several reviews have been focused on specific modifications of thin-film nanocomposite (TFC-RO) membranes for desalination.  In those contributions, different nanomaterials such as carbon-based (carbon nanotube, graphene-oxide), silica, halloysite, zeolite, cellulose nanocrystals-based, metal and metal oxides-based (silver, titanium dioxide, zinc oxide, alumina, metal-organic frameworks, even copper), have been cited. Thus, up to now, a few articles on copper-modiﬁed polymeric membranes are presented in these reviews, compared to the diversity of related works existing in the literature. Although, a few cases have been included for TFC-RO and UF to a lesser extent, without deepening into the challenges inherent in the use of copper.  Being our proposal an extensive analysis of incorporation of copper in polymeric membranes for water treatment (MF, UF, NF, FO, RO), including the advantages and limitations of copper. Specifically, for its effective incorporation on these membranes and analyzing the different improved membrane properties by this modification beyond the anti-biofouling effect, a clear difference from the reported reviews can be underlined. Moreover, our proposal includes strategies for solving the inherent hinders to the specific modifications with copper and perspectives about scale up implementation of these modified membranes. Hence, this work should be interesting to scientists, engineers, and researchers in academy and industry alike. |
| --- |

# Review Question

The team has proposed the following review questions:

- Is the modification of polymeric membranes with copper materials a novel topic?

- What types of polymeric membranes for water treatment have been modified with copper-based materials?

- What are the major benefits obtained from the modification of polymeric membranes with copper- based materials?

- Which techniques are the most frequently used for the modification of polymeric membranes with copper?

- What are the advantages and limitations of these modification techniques for the effective incorporation of copper?

- Which are the current efforts in the modification of polymeric membranes with copper?

- What are the perspectives a future challenges for scaling up copper modified membranes for water treatment?

# Search Strategy

| **Databases**  *List the bibliographic databases to be searched.* |
| --- |
| - WoS - SCOPUS - ScienceDirect - WOL - Google Scholar - Mendeley |
| **Hand Searching**  *List journals or websites that will be hand searched for relevant articles.* |
| Particularly, hand searching was not done for this review article. However, the search methodology led to the identification of a series of important journals in the area from which many of the cited reports were found. For example, Desalination, Journal of Membrane Science, Membranes, Separation and Purification Technology, Applied and Environmental Microbiology, Environmental Science and Technology, Journal of Colloid and Interface Science, Nanotechnology, RSC Advances, among others. |
| **Experts or Stakeholders**  *If experts or key stakeholders are being contacted for additional grey literature or research, list them and how they will be contacted.* |
| Experts or key stakeholders were not contacted for additional grey literature or research |
| **Reference Searches**  *If forward or backward citations will be performed (also known as chain or snowball searching), detail them here.* |
| Forward or backward citation were not performed. |

# Eligibility Criteria

*Operationalize your PICO (or other framework) concepts by explicitly stating what would and would not meet inclusion. Wherever possible, provide definitions, ICD codes or other identifiers to be as clear as possible.*

PICO analysis is not applicable for this review type. However, the eligibility criteria were stablished using the PRISMA flow diagram (see this flow diagram in the attached document). The eligibility criteria stablished were as follows:

**a.- Publication period:** Only articles between 2010 and 2020 were considered. Because from 2010, the first reports about the modification of polymeric membranes with copper materials for water treatment were published.

**b.- Keywords:** copper nanomaterials, polymeric membranes, biofouling, water treatment, nanocomposites. The articles that did not match all keywords used for the search were excluded. For example, they were excluded:

- The modification on the ceramic membranes and cellulose acetate membranes.

- The modified membranes with different applications to the water treatment. For instance, bioreactor with electric fields, among others.

- Modified membranes with other antimicrobial material instead of copper materials.

- The membranes that could not be classified in the following filtration processes: reverse osmosis, forward osmosis, nanofiltration, ultrafiltration, microfiltration.

# Data Extraction

*Provide a description of methods used to collect data from included studies (e.g. categories of data you intend to collect, how many people will conduct extraction, how disagreements will be resolved, etc).*

| The categories for data collection accorded by the work team are the following:   - Polymeric membrane type according to filtration process: Reverse osmosis (RO), nanofiltration (NF), forward osmosis (FO), ultrafiltration (UF) and microfiltration (MF). - Copper materials used to membrane modification: metallic and oxides nanoparticles, salts, composites, metal-polymer complex, and coordination polymers. - Membrane modification approaches to incorporate copper. For instance, the incorporation during the membrane synthesis process or its surface modification using physical and chemical surface modification techniques.   For this, at least four of the co-authors of this manuscript carried out the collection and analysis of the state of the art for each of the different sections presented, based on their areas of expertise. The search and analysis methodology was the same for all cases and the general discussion was centralized by the corresponding authors. |
| --- |

# Study Quality Assessment

*If applicable, describe the tool(s) you will use to assess risk of bias.*

| It is not applicable. |
| --- |

# Data Synthesis

*Describe how you will analyze and summarize the included study results.*

| The included studies results have been analyzed and summarized according to the following steps:  1.- A preliminary section “Copper Overview” that describes the properties, toxic mechanism and relevant features of Copper-based materials. After, the articles are included in a section “Polymeric membranes modified by copper incorporation”. Those have been classified according to the polymeric membrane for each filtration process type (RO, NF, FO, UF, MF). Thus, the aforementioned section has five subsections, one for each filtration process type.  2.- The main results of the selected studies define the base polymer of the modified membrane, the copper material type used to modify the membranes, the modification methods used to incorporate copper material, and performance/ properties of the modified membranes. These details have been extracted and compiled in summary tables by each filtration process.  3.- A brief discussion about the main results of selected studies have been incorporated in each subsection. This discussion has been done by identifying:  a) Changes on different membrane properties such as antibacterial and anti-fouling effect, hydrophilicity increase, improvements of the water flux, the rejection of compounds capacity and structural membrane parameters, and the reduction of concentration polarization phenomena.  b) The advantages and limitations of proposed modifications with insights towards a possible industrial applicability.  c) The relevant detrimental consequences produced by copper incorporation in polymeric membrane.  d) The modification strategies that allow an effective copper incorporation on these polymeric membranes for solving the detrimental consequences previously identified. |
| --- |

# Project Timetable

*Fill out the following Gantt chart according to your estimated project timelines.*

| Year 2020 | January | February | March | April | May | June | July | August | September | October | November | December |
| --- | --- | --- | --- | --- | --- | --- | --- | --- | --- | --- | --- | --- |
| **Preparation** |  |  |  |  |  |  |  |  |  |  |  |  |
| **Conduct searches** |  |  |  |  |  |  |  |  |  |  |  |  |
| **Pilot test eligibility criteria** |  |  |  |  |  |  |  |  |  |  |  |  |
| **Ti/Ab + Full Text Selection** |  |  |  |  |  |  |  |  |  |  |  |  |
| **Pilot test data collection** |  |  |  |  |  |  |  |  |  |  |  |  |
| **Data collection** |  |  |  |  |  |  |  |  |  |  |  |  |
| **Conduct synthesis & interpret results** |  |  |  |  |  |  |  |  |  |  |  |  |
| **Write manuscript** |  |  |  |  |  |  |  |  |  |  |  |  |

# Research Team Member Roles

*Describe the different tasks on the review and who will be responsible for what.*

| Task | Description | Team Member Responsible |
| --- | --- | --- |
| Visualization, supervision, writing-reviewing and editing. | Author creator of the idea for review writing.  Writing-reviewing and editing related to the entire manuscript. | Andreina García |
| Searching- writing-reviewing and editing. | Searching and Writting related to the section “Polymeric membranes modified by copper incorporation-Reverse Osmosis (RO) membranes RO”. Reviewing and editing related to others sections of the manuscripts. | Bárbara Rodríguez |
| Searching- writing-reviewing and editing. | Searching and Writting related to the sections “Polymeric membranes modified by copper incorporation-Forward Osmosis (FO) membranes-Nanofiltration (NF) membranes”. Reviewing and editing related to others sections of the manuscripts. | Hugo Giraldo |
| Searching-writing-original draft. | Searching and Writting related to the section “Polymeric membranes modified by copper incorporation-Ultrafiltration (UF) membranes”. | Yurieth Quintero |
| Searching- writing-original draft. | Searching and Writting related to the section “Overview of Copper-Properties and toxic mechanism”.  Assistance for the elaboration of Prisma flow diagram. Editing of the included figures. | Rodrigo Quezada |
| Searching- writing-original draft. | Searching, Writting and reviewing related to the section “Overview of Copper-Properties and toxic mechanism- Copper-based materials and relevant features” | Natalia Hassan |
| writing-reviewing and editing. | Writing-reviewing and editing related to the section “New challenges and perspectives-Scale up implementation and environmental impact” | Humberto Estay |

# References

| This Review Protocol was created by Sarah Visintini, Maritime SPOR SUPPORT Unit and adapted from the following resources:  Cochrane Public Health Group. (2011) Guide for developing a Cochrane protocol. Retrieved from: <http://ph.cochrane.org/sites/ph.cochrane.org/files/uploads/Guide%20for%20PH%20protocol_Nov%202011_final%20for%20website.pdf>.  Dartmouth Biomedical Libraries. (2012). Systematic Review Steps. Retrieved from [http://www.dartmouth.edu/~library/biomed/services/lgr/docs/SR-Steps-Roles-revised.docx](http://www.dartmouth.edu/%7Elibrary/biomed/services/lgr/docs/SR-Steps-Roles-revised.docx)  Durham University Community. (2009). Template for a Systematic Literature Review Protocol. Retrieved from <https://community.dur.ac.uk/ebse/resources/templates/SLRTemplate.pdf>.  Warwick Medical School. (n.d.) Protocol Template: Systematic Review. Retrieved from <http://www2.warwick.ac.uk/fac/med/staff/bridle/sr/protocol_template.doc>.  World Health Organization. (2011). Review Protocol Template. Retrieved from <http://www.who.int/hrh/education/Rec1_CPDforfacultyteachingstaff.pdf>. |
| --- |
