# Supplementary material for: Copper-Modified Polymeric Membranes for Water Treatment: A Comprehensive Review
Source: Membranes (Basel). 2021 Jan 28;11(2):93. doi: 10.3390/membranes11020093 (PMC7911616; doi:10.3390/membranes11020093)
Supplement: Supplementary file 1 [file membranes-11-00093-s001.zip › membranes-1085993-supplementary/Supplementary Information/PRISMA 2009 flow diagram_AG.doc]

**PRISMA 2009 Flow Diagram**

**Screening**

**Included**

**Eligibility**

**Identification**

Additional records identified through other sources
(n = 511 (Scholar) + Mendeley (49)) )

Records after duplicates removed
(n = 504)

Records screened
(n = 504)

Records excluded
(n = 448)

Records identified through database searching
(n = 20(WoS)+16(SCOPUS) +349 (ScienceDirect)+ 278 (WOL))

Full-text articles assessed for eligibility
(n = 56)

Full-text articles excluded, with reasons
(n = 4)

Studies included in qualitative synthesis
(n = 52)

The eligibility criteria stablished were as follows:

**a.- Publication period:** Only articles between 2010 and 2020 were considered. Because from 2010, the first reports about the modification of polymeric membranes with copper materials for water treatment were published.

**b.- Keywords:** copper nanomaterials, polymeric membranes, biofouling, water treatment, nanocomposites. The articles that did not match all keywords used for the search were excluded. For example, they were excluded:

- The modification on the ceramic membranes and cellulose acetate membranes.

- The modified membranes with different applications to the water treatments. For instance, bioreactor with electric fields, among others.

- Modified membranes with other antimicrobial material instead of copper materials.

- The membranes that could not be classified in the following filtration processes: reverse osmosis, forward osmosis, nanofiltration, ultrafiltration, microfiltration.
